# Synergistic fusion: An integrated pipeline of CLAHE, YOLO models, and advanced super-resolution for enhanced thermal eye detection

**DOI:** 10.1371/journal.pone.0328227

**Published:** 2025-07-18

**Authors:** Persiya J., Sasithradevi A

**Affiliations:** 1 School of Electronics Engineering, Vellore Institute of Technology Chennai, India; 2 Centre for Advanced Data Science, Vellore Institute of Technology Chennai, India; Purdue University, UNITED STATES OF AMERICA

## Abstract

Accurate eye detection in thermal images is essential for diverse applications, including biometrics, healthcare, driver monitoring, and human-computer interaction. However, achieving this accuracy is often hindered by the inherent limitations of thermal data, such as low resolution and poor contrast. This work addresses these challenges by proposing a novel, multifaceted approach that combines both deep learning and image processing techniques. We first introduce a unique dataset of thermal facial images captured with meticulous eye location annotations. To improve image clarity, we employ Contrast Limited Adaptive Histogram Equalization (CLAHE). Subsequently, we explore the effectiveness of advanced YOLO models (YOLOv8 and YOLOv9) for accurate eye detection. Our experiments reveal that YOLOv8 with CLAHE-enhanced images achieved the highest accuracy (precision and recall of 1, mAP50 of 0.995, and mAP50-95 of 0.801), the YOLOv9 model also demonstrated excellent performance with a precision of 0.998, recall of 0.998, mAP-50 of 0.995, and mAP50-95 of 0.753. Furthermore, to enhance the resolution of detected eye regions, we investigate various super-resolution techniques, ranging from traditional methods like Bicubic interpolation to cutting-edge approaches like generative adversarial networks (BSRGAN, ESRGAN) and advanced models like Real-ESRGAN, SwinIR, and SwinIR-Large with ResShift. The performance of these techniques is evaluated using both objective and subjective quality measures. Overall, this work demonstrates the effectiveness of our proposed pipeline, which seamlessly integrates image enhancement, deep learning, and super-resolution techniques. This synergic fusion significantly improves the contrast, accuracy of eye detection, and overall resolution of thermal images, paving the way for potential applications across various fields.

## Introduction

Thermal imaging is a promising technique that offers contact-free and reliable detection of facial features like eyes, crucial for various applications such as security, human-computer interaction, the automotive industry, healthcare, search and rescue, emotion recognition, electrical, and occupational safety [1–3]. Unlike traditional face recognition methods that struggle in low-light or obscured conditions, thermal imaging, with its unique ability to sense infrared radiation, unlocks new possibilities for various technologies [4]. Yawen Lu et al proposed an alternative to LiDAR for nighttime depth estimation using a single thermal image making it a viable solution for nighttime autonomous navigation [5]. These infrared thermal images were created using infrared thermal cameras, which can detect the heat distribution on a target object’s surface [6]. Thermal images obtained using thermal cameras are called thermograms [7]. Various thermal cameras offer a range of color palettes like Iron, Gray, Rainbow, Arctic, Lava, and more [8]. In this article, thermal images used are in the Rainbow color palette. This palette assigns colors to different temperature levels, with cooler areas in blues and greens and warmer regions in yellows and reds. The choice of color palette can significantly impact the interpretation of thermal data and is often tailored to specific applications and preferences. Enhancing thermal images is crucial due to their tendency to suffer from blurring, yet it’s essential to implement enhancement techniques carefully to preserve the temperature distribution information without sacrificing details. Several methods have been introduced in the past to improve the contrast of thermal images, aiming to make details more discernible and enhance overall visibility [9]. The enhancement of thermal images is important for extracting detailed information from low-resolution data, improving object recognition, and enhancing overall image clarity. This process aids in various applications where precise thermal analysis is vital.

The review of the literature reveals a multitude of detection techniques utilizing thermal imagery across a range of applications. To diagnose ocular surface eye disorders, Padmapriya et al. developed YOLOv2-based eye localization of thermal images [10]. Ilikci et al. suggested utilizing the YOLOv3 algorithm to recognize emotions and faces in thermal images [11]. Ghourabi et al [12] employed the YOLOv5 algorithm to identify the eye region from the thermal image to pinpoint the precise temperature in the canthus of the eye and identify any infections that might be present. Ghourabi et al. [13] used YOLOv7 for eye recognition to compute eye canthus temperature in the elderly. Using machine learning and the Internet of Things (IoT), Klaib et al. [14] discussed a range of algorithms that tracked the human eye for various applications, particularly for the elderly and human-computer interaction. A method to identify the face and eye from a thermal image was created by Budzan et al. utilizing a mix of template matching expertise and a modified Randomised Hough Transform (RHT) [15]. Fast eye detection in thermal pictures was carried out by Knapik et al. utilizing a bag-of-visual-words technique with clustering [16]. Zijie Zhou et al. explored driver vigilance detection using thermal imaging, integrating YOLOv5 for facial region detection and a hybrid-attention deep learning model for classification [17]. Various deep learning-based algorithms have been proposed to detect and localize the face and eyes in thermal images [18]. However, due to blurred edges and lower quality compared to visible-light images, the process of enhancing eye resolution in thermal images is more complicated. In the realm of facial recognition with low-resolution thermal images, a texture-based detector is applied, employing Haar features and the AdaBoost algorithm. Subsequently, the interplay among these facial characteristics is analyzed by employing a complex Gaussian distribution, which remains unaffected by rotation [19]. In the context of Child-Robot Interaction, a system for emotion recognition in children has been proposed, which records facial images using both visual and Infrared Thermal imaging [20]. Many deep learning architectures [21] have been explored by researchers to enhance the resolution of thermal images. GAN-based methods were used to enhance and restore the resolution of thermal images [22]. Researchers have found that customizing guiding information for thermal imaging yields superior results, especially within the framework of guided super-resolution [23]. This system could potentially be adapted to enhance the resolution of eye images.

Thermal images inherently suffer from low resolution and poor contrast due to the nature of infrared sensors and the thermal radiation they detect [19]. This limitation is primarily due to the longer wavelengths of thermal radiation compared to visible light, leading to less distinct edges and finer details [9]. Thermal images typically exhibit poor contrast because the temperature differences between objects are often minimal, causing the images to appear flat and less informative. These challenges can hinder the accuracy and effectiveness of thermal imaging, necessitating advanced image processing techniques to enhance the quality and extract meaningful information [24]. While deep learning-based models leverage reference images and feature fusion for enhanced quality assessment [25,26], our study focuses on a more practical non-reference approach. Given the absence of reference images in thermal imaging, we evaluate the quality of super-resolved thermal images using well-established non-reference image quality assessment metrics along with subjective Mean Opinion Scores to ensure a comprehensive assessment.

Existing research articles typically address the improvement of image quality by focusing on one specific aspect, such as detection, enhancement, or super-resolution. Each of these approaches has its own merits, contributing to either better image resolution or enhanced contrast, but they are generally applied in isolation. However, in our work, we have developed a hybrid model that combines these techniques into a single, integrated approach. By simultaneously incorporating enhancement, detection, and super-resolution methods, our model provides a more holistic solution to image quality improvement. This comprehensive strategy ensures that we achieve both higher contrast and superior resolution at the same time, rather than prioritizing one over the other. The integration of these techniques allows for more effective and efficient image processing, resulting in clearer, more detailed thermal images that can be crucial for accurate analysis and interpretation. Our research focuses on creating a highly accurate and efficient system for locating eyes in human faces using infrared thermal images. The research aims to overcome the inherent limitations of thermal images, namely their low resolution and poor contrast. The process involves curating and annotating a large database of thermal face images, enhancing image quality with CLAHE (Contrast Limited Adaptive Histogram Equalization), and employing various YOLO versions to locate eye regions. After eye identification, the importance of high-resolution thermal images is emphasized, prompting the exploration of super-resolution techniques. These methods aim to enhance spatial resolution and address the common challenge of low-resolution thermal imaging. Our proposed work to detect the human eyes on thermal images and enhance the resolution includes the following novel contributions:

- Multifaceted novel pipeline for accurate eye detection in thermal images, addressing low resolution and poor contrast.
- Introduced a unique dataset with labelled eye locations for training and evaluation.
- Pre-processed thermal images with CLAHE for improved contrast and boost eye detection.
- Demonstrated the effectiveness of YOLO models (especially YOLOv8 and YOLOv9) for improved detection accuracy.
- Evaluated super-resolution techniques to enhance the resolution of detected eyes.

This paper is organized into seven sections. Section 2 introduces the thermal image dataset. Section 3 provides a brief idea about the methodology and the algorithms used. Section 4 provides an in-depth discussion of the performance analysis and results achieved for different detection and super-resolution algorithms. Section 5 discusses the practical implications and potential applications. Section 6 outlines the limitations and suggests future improvements. The conclusions drawn from the observations are presented in Section 7.

## Materials-dataset details

There is a significant shortage of high-quality thermal human face image datasets for eye detection. Existing datasets primarily focus on thermal emotion recognition and pedestrian detection, with many researchers relying on private datasets that are not publicly accessible. This highlights the urgent need for a standardized thermal human face image dataset. To address this gap, we created a dataset using a FLIR-E75 thermal imaging camera, capturing images at a resolution of 640x480 pixels. The thermal images were collected from 308 volunteers at Vellore Institute of Technology, Chennai, including 50 females and 258 males aged 20–40, all of whom provided proper consent. Volunteers include students, research scholars, and faculty members.

This study received approval from the Ethics Committee at Vellore Institute of Technology, Chennai. The dataset was collected over a period of two months, spanning from June 3, 2024, to July 31, 2024. The data collection adhered strictly to the ethical principles outlined in the Declaration of Helsinki for research involving human subjects. Participants were verbally informed about the research’s nature and everyone signed a consent form authorizing the use of their data for academic and research purposes. Participants under the age of 18, including children, were excluded from the study. The consent form included a concise explanation of data collection details. In accordance with the guidelines and format provided by the Institutional Ethics Committee for Studies on Human Subjects (IECH) at Vellore Institute of Technology, Chennai, the consent form was detailed, and participants’ written consents were collected. All consent forms have been securely stored, and the collected image data has been anonymized. Data was collected in a controlled environment to ensure standardization and support various applications, including healthcare monitoring of eye temperature.

Participants were positioned one meter from the camera. Precise annotation is crucial for supervised learning in eye detection, so each image was meticulously annotated with bounding boxes around the eye regions by well-trained researchers and reviewed by two teams of computer vision experts for accuracy. The dataset is divided into three groups: 70% for training, 20% for validation, and 10% for testing, ensuring robust evaluation of the model’s performance. The self-curated dataset aims to advance research in thermal eye detection by providing a valuable resource for the scientific community.

**Fig 1:**
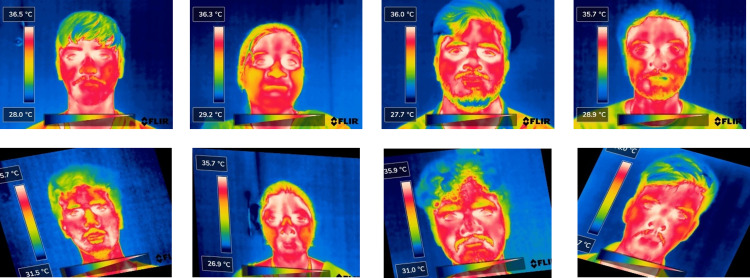
Sample images used & augmented images used. The first row represents the original thermal images captured using a FLIR camera. The second row shows augmented versions of these images, incorporating transformations such as rotation, scaling, and vertical shear distortions.

Augmentation enriches the dataset by introducing diverse variations, enabling deep learning models to generalize better across different scenarios and mitigate overfitting. It enhances model robustness by simulating real-world variability, leading to more reliable and adaptable performance. The curated dataset of 308 images is subjected to various augmentation steps. This augmentation includes flipping the images horizontally and vertically, performing various rotations (90 degrees clockwise, counter-clockwise, and upside down), introducing random rotations between −20 and 20 degrees, and applying horizontal and vertical shear distortions of up to 15 degrees. Each original image undergoes augmentation to produce approximately eight distinct images. In total, 2318 images are generated through this augmentation process. Flipping, rotation, and shear distortions are utilized to augment the images because these augmentation techniques preserve the pixel values and the temperature information encoded in the thermal images. They do not change the pixel values or add any noise. Table 1 summarises the number of images in the dataset. Sample thermal images of the dataset are depicted in Fig 1.

**Table 1:** Dataset Details.

	Total Number of images	Train images	Validation images	Test images
Raw images	308	216	61	31
After Augmentation	2318	1629	458	231

*Note:* The dataset initially contained 308 raw images, which were split into training, validation, and test sets. After applying augmentation techniques (such as rotation, flipping, and vertical shear distortions), the dataset was expanded to 2318 images while maintaining the same train-validation-test split ratio. Augmentation enhances model robustness and generalization by introducing variations in the dataset.

## Methodology and algorithms used

**Fig 2:**
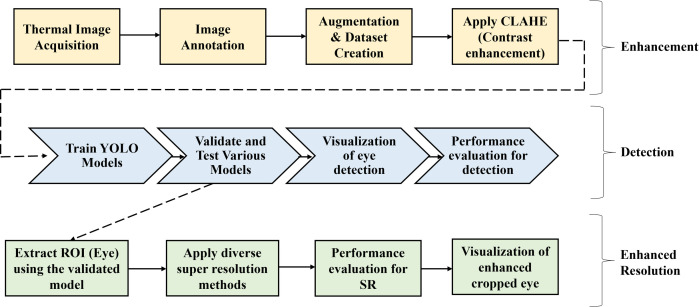
Block diagram of the proposed work. The block diagram outlines the workflow for eye detection and enhancement from thermal images. It includes image acquisition, annotation, augmentation, contrast enhancement, YOLO-based detection, ROI extraction, super-resolution application, and performance evaluation.

The first step is thermal image acquisition, which is the most critical phase in this methodology. The captured images are then labeled, annotated, and augmented to create a dataset. This dataset is subsequently divided into training, validation, and testing sets. The proposed methodology’s flow diagram is illustrated in Fig 2. Two sets of experiments are conducted to evaluate how well different YOLO algorithms perform in detecting eye regions in thermal images of human faces. In the first experiment, the YOLO algorithms are applied directly to the raw dataset. In the second experiment, the YOLO algorithms are applied to images that have been enhanced using various image processing techniques. Once a YOLO model has been selected, it is used to detect the eye regions in the thermal images. The Region of Interest (ROI) that is the eye regions are then cropped from the images based on the identified coordinates. Due to the cropping process, the resolution of the eye regions is low. To address this, various super-resolution algorithms are employed to enhance the resolution of the cropped eye regions. The performance of these super-resolution algorithms is then evaluated. Algorithm I provides a detailed breakdown of the steps involved.

**Table:** 

Algorithm I	
*Input:* Self–created thermal dataset in.jpg *Output:* Resolution enhanced detected eye region	
1	*Preprocess the images*: - Manually annotate and randomly split the dataset into train (70%), validation (20%), and test (10%) sets. - Apply augmentations, including • Flipping (horizontal and vertical). • Rotations (90 degrees clockwise, 90 degrees counterclockwise, Upside down). • Random rotations (Between −20 degrees and +20 degrees). • Shear (Horizontal shear ±15 degrees, Vertical shear ±15 degrees). - Resize the images to 640x640.
2	*Enhancement*: - Use CLAHE by conducting an ablation study by on CLAHE parameters (Clip Limit, Grid size) to determine optimal settings.
3	YOLO Model Training - Apply YOLOv8 and YOLOv9 models to both raw images and CLAHE-enhanced images. - Set the hyperparameters and train models using pre-trained weights. - Validate and test the trained models using metrics like Precision, Recall, mAP50, mAP50-95.
4	*Performance Evaluation* - Evaluate the performance of all YOLO models and compare their detection accuracy on both raw and enhanced images.
5	*Crop the ROI*: - Based on the detected bounding box coordinates.
6	*Apply Super-Resolution Algorithms:* Apply various super-resolution algorithms: *Bicubic interpolation, BSRGAN, ESRGAN, Real ESRGAN, SwinIR, SwinIR-Large and ResShift*
7	*Performance analysis of super-resolution algorithms:* *-* Analyze the performance of all super-resolution algorithms using both objective and subjective assessment.
8	*Final Hybrid Model:* Combine the best-performing YOLO model for eye detection with the best-performing super-resolution algorithm to create a final model that detects and enhances the resolution of eye regions in thermal images.

### Enhancement using CLAHE

To improve the contrast of thermal face images, Local Histogram Equalization (LHE) with Bilateral Filtering is employed. LHE with Bilateral Filtering, a more sophisticated approach, enhances contrast locally while preserving details [27]. It first converts the image to LAB color space and applies bilateral filtering to the luminance channel (L) to smooth intensity variations while maintaining edges. Subsequently, LHE is applied to small image patches within the filtered L channel, enhancing contrast localized to specific regions. Finally, the processed L channel is merged with the original A and B channels, and the image is converted back to its original color space. This combined approach ensures improved visibility of temperature variations in human faces within the thermal images.

CLAHE, which stands for Contrast Limited Adaptive Histogram Equalization, is an image processing technique employed to enhance the contrast of thermal images. CLAHE is an extension of LHE that addresses the over-amplification of noise issues. It limits the amplification by clipping the histogram at a specified value (clip limit) before performing histogram equalization in each patch. By dividing the thermal image into tiles or blocks, CLAHE ensures local contrast improvement while preventing noise exaggeration associated with global equalization. The degree of contrast enhancement allowed in each tile is controlled by a parameter known as the contrast limiting factor. This factor regulates the process by clipping histogram bins that exceed a specified threshold, thus avoiding the exaggeration of noise or extreme contrast in those areas [28]. When applying CLAHE to thermal images, it is often done within the context of the LAB color space. This choice is advantageous because the LAB color space separates color information from the thermal intensity (brightness) information. This separation allows CLAHE to boost contrast while preserving the essential thermal details. An ablation study was conducted to investigate the effects of different combinations of ClipLimit and GridSize parameters. This study involved testing various settings, including (ClipLimit = 2, GridSize = 4x4), (ClipLimit = 2, GridSize = 8x8), (ClipLimit = 4, GridSize = 16x16), and (ClipLimit = 10, GridSize = 32x32). The application of CLAHE to thermal images finds wide utility in various fields, including thermal surveillance, medical thermography, and other areas where improved contrast is essential for accurate visual interpretation and analysis.

### Detection techniques using YOLOv8 and YOLOv9

YOLO (You Only Look Once) creates a grid out of the image. Certain numbers, such as class probabilities and bounding box parameters, are computed for each grid. Additionally, a class prediction is based on every cell. YOLO performs well on objects of different sizes because it uses multi-scale feature maps to gather global context data. The algorithm is also skilled at locating tiny items, which can be difficult for other detection techniques [29]. YOLO has gone through several evolutions from YOLOv1 to recent YOLO version 9 being major developments that have further enhanced its accuracy and performance. The YOLO family of models has three main blocks, they are backbone, neck, and head. YOLO has been modified to do specialized tasks including face detection, position estimation, and even eye recognition in infrared thermal photos of human faces. This work examines YOLO’s adaptability beyond basic object detection. YOLO’s influence on the direction of object detection is still very important as it develops and motivates new computer vision research. Each iteration introduces novel features aimed at improving accuracy and performance. YOLOv5 incorporates CSPDarknet53 and CSP-PANet techniques [30], enhancing eye detection accuracy through feature map splitting and aggregation. Where, CSP stands for Cross Stage Partial connections. YOLOv5 computes the class loss and objectness loss using Binary Cross Entropy (BCE), while location loss is determined using CIoU (Complete Intersection over Union) loss. These three components collectively shape the YOLO loss function, as depicted in Equation 1.


$$Yolo5\_Loss=\lambda_{1}\;L_{cls}+\lambda_{2}\;L_{obj}+\lambda_{3}\;L_{loc}\;\;\;\;\;\;$$ (1)

YOLOv7, inspired by ELAN (Efficient Layer Aggregation Network) architecture, integrates Feature Pyramid Network (FPN) and BoF techniques for superior eye localization without relying on pre-trained backbones [31].

#### YOLOv8 architecture.

**Fig 3:**
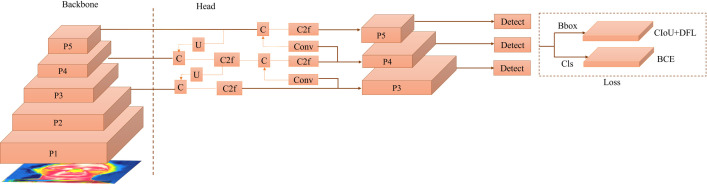
YOLOV8 Architecture. The YOLOv8 architecture follows a multi-scale feature extraction approach. The backbone processes input images through different feature levels (P1–P5). ‘C’ denotes concatenation, ‘C2f’ represents a modified CSP layer integrating two parallel gradient flow branches, and ‘U’ indicates upsampling. The detection head outputs bounding boxes (Bbox) optimized using CIoU-DFL and classification scores (Cls) using BCE loss.


The latest iteration of YOLO, known as YOLOv8, maintains the same architectural foundation as its predecessors but introduces several significant enhancements for precise eye localization in thermal human face images. Let the input image be denoted by: $x\in {\mathbb{R}}^{H\times W\times C}$, where H is the height, W is the width, and C is the number of channels. Illustrated in Fig 3, YOLOv8 integrates the Path Aggregation Network (PAN) [32] and FPN, along with an innovative labeling tool to streamline the annotation process. The backbone network extracts feature maps from the input image at various levels. The feature extraction process involves multiple convolutional layers. FPN progressively reduces the spatial resolution of input images while increasing the number of feature channels, generating feature maps capable of detecting eyes at various scales and resolutions. Specifically, the FPN creates a pyramid structure by combining high-resolution low-level features with low-resolution high-level features. The output of the FPN is expressed as in equation 2.

$$P_{l}=Conv\left(lateral\left(P_{l+1}\right)\right)+\;P_{l}\;$$ (2)

Where $P_{l}$ denotes the feature map at the level l. Conv performs convolution operations on the lateral Connections. Conversely, PAN employs skip connections to merge features from multiple network layers, enhancing adaptability and effectiveness due to its anchor-free detection approach. YOLOv8 incorporates a modified CSP layer, referred to as the C2f module, which combines Concatenation (C), Upsampling (U), and the C2f module to elevate eye detection accuracy [33]. The C2f module is mathematically represented in equation 3.


$$C2f=Concat\left(Upsample\left(F\right),\;F_{prev}\right)$$ (3)

Where F denotes the current feature map and $F_{prev}$ denotes the previous feature map. For bounding box loss, YOLOv8 employs Distribution Focal Loss (DFL) and Complete Intersection over Union (CIoU) loss functions, while classification loss relies on binary cross-entropy. The loss function is given in Equation 4.


$$Loss=\frac{\lambda_{box}}{N}L_{box}\left(\theta \right)+\;\frac{\lambda_{cls}}{N}L_{cls}\left(\theta \right)+\;\frac{\lambda_{dfl}}{N}L_{dfl}\left(\theta \right)+\;\phi$$ (4)

Where $\lambda_{box}$, $\lambda_{cls}$, $\lambda_{dfl}\;$ are the weights for box loss, class loss, and Distribution focal loss. And ϕ and N represents the weight decay and number of cells that contain the object. The result is an impressive combination of speed and precision, making YOLOv8 a powerful choice for eye localization in thermal human face images [34].

#### YOLOv9 architecture innovations and improvements.

YOLOv9, the latest iteration in the YOLO series, stands out for its exceptional performance, efficiency, and innovative techniques that address key challenges in deep learning, particularly information loss during training. To combat this, YOLOv9 introduces Programmable Gradient Information (PGI), employing an auxiliary reversible branch to ensure information preservation and reliable gradient generation [35]. PGI is designed to preserve crucial information and ensure reliable gradient generation during the training process. It employs an auxiliary reversible branch that works in conjunction with the main network. The reversible branch helps in backward propagation to maintain gradient flow and prevent vanishing gradients. This auxiliary pathway is expressed in Equations 5, 6, 7, and 8.

Forward Pass:


$$x_{out}=f\left(x_{in}\right)$$ (5)

Where $x_{in}$ is the input to the network and f is the transformation applied by the network layers.

Reversible auxiliary branch:


$$y_{out}=g\left(x_{in}\right)$$ (6)

$$z_{out}=h\left(y_{out}\right)$$ (7)

Where g and h are auxiliary functions that preserve information.

Gradient calculation:


$$\frac{\partial L}{\partial x_{in}}=\;\frac{\partial L}{\partial x_{out}}+\;\frac{\partial L}{\partial y_{out}}+\;\frac{\partial L}{\partial z_{out}}\;$$ (8)

Where L is the loss function. This auxiliary pathway enhances the model’s learning process, enabling effective extraction of crucial insights even with lightweight architectures. Additionally, YOLOv9 incorporates the Generalized Efficient Layer Aggregation Network (GELAN) to efficiently merge features from different network levels, crucial for accurate detection of objects of various sizes and scales. Unlike conventional methods, GELAN utilizes conventional convolutions to achieve better use of parameters without sacrificing efficiency. The GELAN process of feature extraction and feature aggregation is represented in Equations 9 and 10 respectively.


$$F_{i}=\;Conv_{i}\left(x\right)$$ (9)

Where $Conv_{i}$ represents the conventional convolutional operation at the layer i and x is the input image.


$$F_{agg}=\;\sum_{i=1}^{n}\alpha_{i}F_{i}$$ (10)

**Fig 4:**
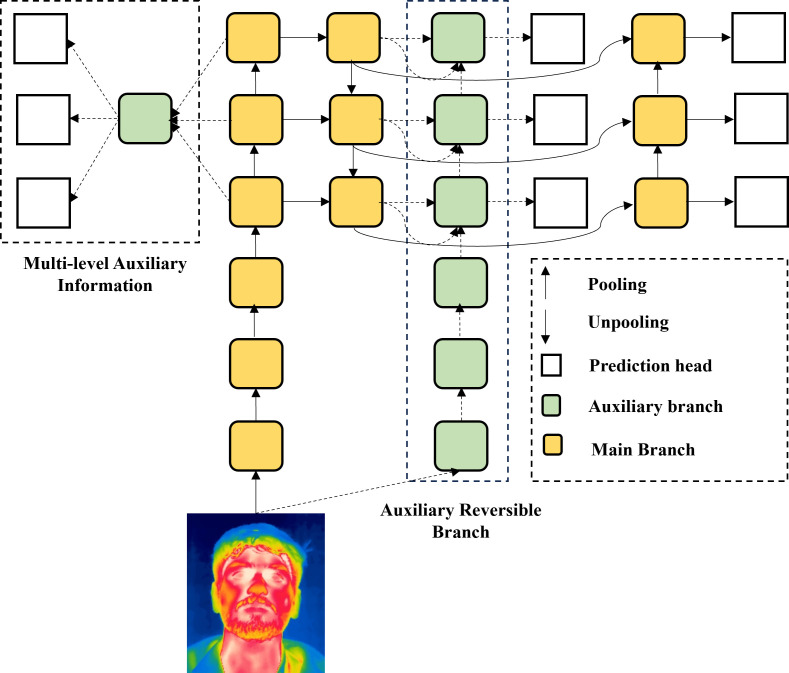
Programmable Gradient Information (PGI). An auxiliary reversible branch that preserves gradient flow, preventing information loss and enhancing feature extraction.

**Fig 5:**
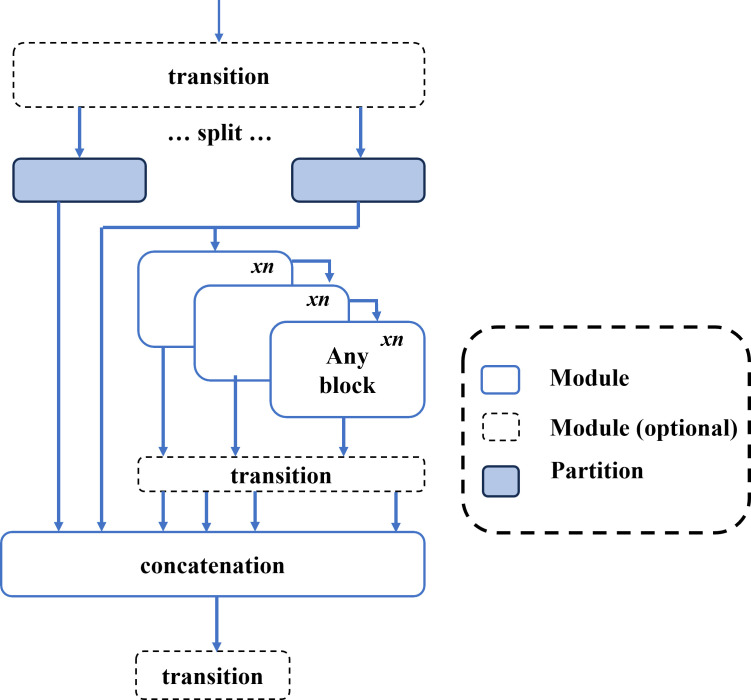
Architecture of Generalized Efficient Layer Aggregation Network (GELAN). A hierarchical structure that efficiently merges multi-scale features using conventional convolutions for improved object detection.


Where $\alpha_{i}$ are learnable weights and $F_{agg}$ is the aggregated feature map. Figs 4 and 5 illustrate the two key innovations within YOLOv9’s architecture: PGI and the GELAN. These components work together to enhance the model’s performance. Furthermore, YOLOv9 adopts a novel mosaic data augmentation technique, randomly combining four images into a single training image to create a diverse and realistic dataset, thereby enhancing generalization and reducing overfitting. The mosaic augmentation is described in Equations 11 and 12 respectively.

$$I_{mosaic}=Combine\left(I_{1},I_{2},I_{3},I_{4}\right)$$ (11)

Where $I_{1},I_{2},I_{3},I_{4}$ are the four images to be combined.


$$I_{final}=Crop\left(Position\left(I_{mosaic}\right)\right)$$ (12)

Where Position arranges the images in a grid and Crop extracts a random region. The overall loss function for YOLOv9 includes contributions from box loss, class loss, and a new term for gradient preservation (GP loss), ensuring robust training. The loss function is expressed as in Equation 13.


$$Loss=\frac{\lambda_{box}}{N}L_{box}\left(\theta \right)+\;\frac{\lambda_{cls}}{N}L_{cls}\left(\theta \right)+\;\frac{\lambda_{gp}}{N}L_{gp}\left(\theta \right)+\;\phi$$ (13)

Where $\lambda_{box}$, $\lambda_{cls}$, $\lambda_{gp}\;$ are the weights for box loss, class loss, and gradient preservation loss. And ϕ represents the number of cells that contain the object. *N* represents the weight decay. While building upon the successes of previous iterations like YOLOv7 and YOLOv8, YOLOv9 represents a distinct evolution within the YOLO family by addressing information loss issues, maintaining efficiency, and introducing innovative techniques like PGI and GELAN for superior object detection performance.

### Super-resolution techniques

Super-resolution techniques are methods employed in image processing to enhance the resolution or quality of an image beyond its original dimensions. Image processing methods and Generative Adversarial Networks (GANs) have significant importance in the development of super-resolution techniques. Bicubic interpolation is a traditional method used for image super-resolution [36]. It is employed in the context of enhancing low-resolution thermal images of the eye to achieve higher-resolution outputs. Traditional methods like bicubic interpolation offer a quick but often blurry solution, while advanced techniques leverage GANs for improved results. These diverse super-resolution techniques cater to providing solutions for enhancing the resolution of thermal images, particularly in eye detection. BSRGAN and ESRGAN [37] are notable GAN-based approaches designed for super-resolution tasks. BSRGAN creates high-resolution thermal eye images even without access to original high-resolution versions. ESRGAN, an improvement over SRGAN, excels in producing visually pleasing and highly detailed high-resolution images, suitable for real-time applications. Real-ESRGAN adopts a unique approach by training purely with synthetic data, enabling it to address a variety of real-world image degradation challenges [38]. Its high-order degradation modeling process provides superior visual performance over diverse datasets. However, the computational demands of training and deploying these models may limit their real-time applicability in certain scenarios. SwinIR and its variant SwinIR-Large utilize transformer-based models to capture long-range dependencies in the data, particularly useful for handling low-resolution thermal images of the eye [39]. By employing stacked Residual Swin Transformer Blocks, these models progressively extract high-level features and complex relationships, achieving high-quality image restoration. SwinIR-Large is a larger and more powerful version of the original model with an increased number of parameters and layers for potentially better performance. Hence requiring more computational resources. ResShift, built on the concept of residual learning, enhances the resolution of low-resolution thermal images by merging information from different network parts while keeping computational demands low [40]. Trained on large datasets, ResShift offers a promising solution for providing clearer and more detailed images of human eyes.

## Results and discussion

The thermal camera generates images with a resolution of 640x480 pixels. For creating ground truth data, manual tagging was performed using an annotation tool. Three sets of images are prepared: a training set, a validation set, and a test set. All these images are resized to 640x640 pixels each. Python is employed as the programming language for coding and execution on a laptop equipped with an Intel Core i5 processor, 64-bit operating system, operating frequency 3.20GHz with 8GB memory. References for the coding and execution processes are drawn from GitHub repositories such as “https://github.com/ultralytics/ultralytics.git”, “https://github.com/WongKinYiu/yolov9” “https://github.com/zsyOAOA/ResShift”, “https://github.com/xinntao/ESRGAN”, “https://github.com/WongKinYiu/yolov7”, “https://github.com/JingyunLiang/SwinIR”. The YOLOv5, YOLOv7, YOLOv8, and YOLOv9 models are trained using the pre-trained weights of YOLOv5s.pt, YOLOv7.pt, YOLOv8s.pt, and gelan-c.pt, respectively. Default model settings, including learning rate, activation function, and optimization techniques are utilized and are depicted in Table 2. Additionally, LHE with Bilateral Filtering, and CLAHE are applied to enhance the thermal images.

**Table 2:** Hyperparameters used in various YOLO models.

Parameters	YOLOv5	YOLOv7	YOLOv8	YOLOv9
Image_size	640x640	640x640	640x640	640x640
Epochs	100	100	100	100
Batch	16	16	16	16
Weights	Yolov5s.pt	Yolov7.pt	Yolov8s.pt	gelan-c.pt
Learning_rate	0.01	0.01	0.002	0.01
Momentum	0.937	0.937	0.9	0.937
Weight_decay	0.0005	0.005	0.0005	0.0005

*Note:* Comparison of key hyperparameters across YOLOv5, YOLOv7, YOLOv8, and YOLOv9, including image size, epochs, batch size, weights, learning rate, momentum, and weight decay. YOLOv9 utilizes GELAN-based weights (gelan-c.pt) for optimized detection performance.

### Performance evaluation metrics

#### Performance evaluation metrics for detection.

Evaluating the system’s performance is crucial to determining its accuracy and resilience in the context of eye detection in infrared thermal photographs of human faces utilizing YOLO. To evaluate the performance of the created eye localization system, several common assessment metrics are frequently employed.

**Precision:** Precision measures the proportion of accurately identified eye regions among all regions that were assumed to be eyes. It is determined by dividing the total of true positive and false positive detections by the proportion of true positive (TP) detections which is given in Equation 14. Low numbers of false alarms are indicative of great precision.


$$Precision=\;\frac{True\;Positive}{True\;Positive+False\;Positive}$$ (14)

**Recall (Sensitivity):** Recall represents the proportion of correctly identified eye regions among all ground truth eye regions. It is sometimes referred to as sensitivity or true positive rate (TPR). The ratio of true positive detections to the total of true positive and false negative (FN) detections is used to compute it and is given in Equation 15. Low missed detection rates are indicated by a high recall rate.


$$Recall=\;\frac{True\;Positive}{True\;Positive+False\;Negative}$$ (15)

**Intersection over Union (IoU):** IoU calculates the amount of space where the predicted and actual bounding boxes overlap. Equation 16 defined IoU. It is determined by dividing the intersection’s area by the union’s area between the expected and actual bounding boxes. Better localization accuracy is indicated by higher IoU values.


$$IoU=\;\frac{Area\;of\;Intersection}{Area\;of\;Union}$$ (16)

Average precision (AP): The area under the precision-recall curve is known as average precision (AP). It lists the model’s effectiveness at each confidence score threshold. The quality of the detections is represented by a single scalar value that AP offers. AP has a value between 0 and 1, with a higher number indicating superior performance.

**mAP (mean Average Precision):** mAP is the average of the AP scores from several classes or categories. It provides an overall assessment of the model’s object detection accuracy by averaging the AP values for all classes. Different mean Average Precision (mAP) metrics, such as mAP50 and mAP50-95, serve various purposes depending on the task, dataset, and the importance you place on precision and recall. If a balance between precision and recall is needed, mAP50 is a suitable choice. For a comprehensive perspective, mAP50-95 offers insights into performance across different Intersections over Union (IoU) thresholds. Ultimately, selecting the right metric hinges on aligning it with the specific application requirements and goals. mAP50 and mAP50-95 are determined in this research article because mAP50 values show better object detection performance at a 50 percent intersection over union (IoU) threshold because they reflect a higher degree of alignment between predicted bounding boxes and ground truth boxes, and mAP50-95 provides an overview of performance across various IoU thresholds.

#### Performance evaluation metrics for super-resolution.

Evaluating the super-resolution step on the detected eye regions is also important. Since we lack high-resolution ground truth images for comparison, we rely on different metrics that don’t require a reference image, along with subjective evaluation of the improved images. Although NIQE, BRISQUE, and PIQE metrics were initially developed for natural images, they have been effectively employed in thermal imaging contexts due to their ability to quantify general perceptual artifacts and image degradation independent of image modality [41,42].

**NIQE (Natural Image Quality Evaluator):** NIQE is a metric designed to assess the quality of natural images without relying on reference images [43]. It quantifies the level of distortion and artifacts present in an image by analyzing statistics related to natural image properties. Lower NIQE scores indicate higher image quality, making it a useful tool for evaluating the perceptual quality of images without the need for reference images.

**BRISQUE (Blind/Referenceless Image Spatial Quality Evaluator):** BRISQUE is a reference-free image quality assessment metric that evaluates the spatial quality of images. BRISQUE analyzes statistical features extracted from an image and uses a trained model to predict the perceived image quality. Lower BRISQUE scores indicate better image quality.

**PIQE (Perceptual Image Quality Evaluator):** PIQE is a perceptual quality assessment metric that aims to measure the perceived quality of images by considering various aspects of human visual perception. It takes into account factors such as contrast, sharpness, and color fidelity to provide a comprehensive evaluation of image quality. PIQE is designed to be sensitive to subtle distortions that may impact the perceived quality of an image. Higher PIQE scores suggest lower perceptual quality.

### Comparative analysis of detection algorithms

Table 3 summarises the ablation study on clipLimit and titleGridSize. Various YOLO models are executed on the self-created dataset without augmentation, employing different combinations of clipLimit and titleGridSize parameters. Based on the study conducted for CLAHE, the parameters are configured to clipLimit = 2.0 and tileGridSize=(8, 8).

**Table 3:** Analyzing the Impact of CLAHE parameter Variations on Different YOLO Models on the dataset without Augmentation.

		Precision	Recall	mAP50	mAP50-95
ClipLimit = 2 GridSize = 4x4	YOLOv5	0.967	1	0.991	0.685
	YOLOv7	0.967	1	0.98	0.629
	YOLOv8	0.967	1	0.99	0.704
	YOLOv9	0.967	1	0.992	0.722
**ClipLimit = 2** **GridSize = 8x8**	**YOLOv5**	**0.999**	**1**	**0.995**	**0.685**
	**YOLOv7**	**1**	**1**	**0.995**	**0.673**
	**YOLOv8**	**0.999**	**1**	**0.995**	**0.733**
	**YOLOv9**	**0.999**	**1**	**0.995**	**0.721**
ClipLimit = 4 GridSize = 16x16	YOLOv5	0.951	1	0.976	0.697
	YOLOv7	0.951	1	0.982	0.673
	YOLOv8	0.95	1	0.958	0.705
	YOLOv9	0.95	1	0.975	0.717
ClipLimit = 10 GridSize = 32x32	YOLOv5	0.983	1	0.993	0.685
	YOLOv7	0.983	0.992	0.988	0.667
	YOLOv8	0.983	1	0.992	0.731
	YOLOv9	0.975	1	0.995	0.730

*Note:* This table evaluates the effect of Contrast Limited Adaptive Histogram Equalization (CLAHE) parameters (ClipLimit and GridSize) on Precision, Recall, mAP50, and mAP50-95 across YOLOv5, YOLOv7, YOLOv8, and YOLOv9. Different ClipLimit and GridSize values influence model performance, with higher ClipLimit and finer GridSize generally improving feature enhancement but affecting overall mAP50-95 consistency. YOLOv8 and YOLOv9 achieve the best overall balance in precision and mAP across different CLAHE settings.

The YOLO models’ performance is assessed on raw images, locally histogram-equalized with bilaterally filtered images, and CLAHE-enhanced images. Table 4 provides a summary and comparison of precision, recall, mAP50, and mAP50-95 metrics for eye localization in thermal images using different YOLO models.

**Table 4:** Performance Metrics Comparison for Various Detection Algorithms.

On raw Thermal images of human faces					
		Precision	Recall	mAP50	mAP50-95
Without Augmentation	YOLOv5	0.98	0.496	0.517	0.353
	YOLOv7	0.968	0.992	0.986	0.641
	YOLOv8	0.935	0.984	0.981	0.674
	YOLOv9	0.98	0.5	0.683	0.476
With Augmentation	YOLOv5	0.997	0.997	0.993	0.783
	YOLOv7	0.968	0.998	0.993	0.608
	YOLOv8	0.998	0.999	0.993	0.787
	YOLOv9	0.998	0.998	0.993	0.795
On LHE + BL enhanced thermal images					
		**Precision**	**Recall**	**mAP50**	**mAP50-95**
Without Augmentation	YOLOv5	1	1	0.995	0.703
	YOLOv7	1	1	0.996	0.679
	YOLOv8	0.999	1	0.995	0.720
	YOLOv9	0.999	1	0.995	0.705
With Augmentation	YOLOv5	0.998	0.997	0.995	0.724
	YOLOv7	0.998	0.998	0.996	0.715
	YOLOv8	1	1	0.995	0.746
	YOLOv9	0.998	0.998	0.995	0.752
On CLAHE-enhanced thermal images					
		**Precision**	**Recall**	**mAP50**	**mAP50-95**
Without Augmentation	YOLOv5	0.999	1	0.995	0.680
	YOLOv7	0.999	1	0.995	0.673
	YOLOv8	0.999	1	0.995	0.7333
	YOLOv9	0.999	1	0.995	0.721
With Augmentation	YOLOv5	1	1	0.995	0.780
	YOLOv7	0.998	0.991	0.995	0.594
	**YOLOv8**	**1**	**1**	**0.995**	**0.796**
	**YOLOv9**	**0.998**	**0.998**	**0.995**	**0.753**

*Note:* This table compares YOLOv5, YOLOv7, YOLOv8, and YOLOv9 on raw, Local Histogram Equalization and Bilateral Filtering (LHE + BL)-enhanced, and CLAHE-enhanced thermal images of human faces, evaluating Precision, Recall, mAP50, and mAP50-95 with and without augmentation. Augmentation significantly boosts performance across all models, with CLAHE improving fine-grained feature extraction. YOLOv8 and YOLOv9 achieve the highest mAP50-95, demonstrating their robustness in thermal image-based detection.

In general, with augmentation, there is an improvement in precision, recall, and mAP scores for most models, indicating that data augmentation is beneficial for these models. YOLOv8 with augmentation particularly stands out with high precision, recall, and mAP scores. The evaluation of YOLOv5, YOLOv7, YOLOv8, and YOLOv9 models on raw thermal images without augmentation reveals notably diminished overall performance. When augmentation is introduced, there is a notable performance improvement, especially for YOLOv5, YOLOv8, and YOLOv9 leading to higher precision, recall, and mAP50-95. A comprehensive comparison across different image enhancement techniques indicates that CLAHE-enhanced thermal images consistently demonstrate superior performance across all metrics, followed closely by LHE+Bilateral Filtering. These techniques consistently outperform raw thermal images in terms of precision, recall, and mAP scores. Moreover, the findings indicate that utilizing CLAHE-enhanced augmented thermal images with the YOLOv8 model yields superior performance compared to other models, achieving high precision (1), recall (1), mAP-50 (0.995), and mAP50-90 (0.796) values. Following closely, the YOLOv5 and YOLOv9 models. The YOLOv9 model attains precision (0.998), recall (0.998), mAP-50 (0.995), and mAP50-95 (0.753). This enhancement enhances our capability to efficiently and accurately identify eyes in thermal images of human faces.

**Fig 6:**
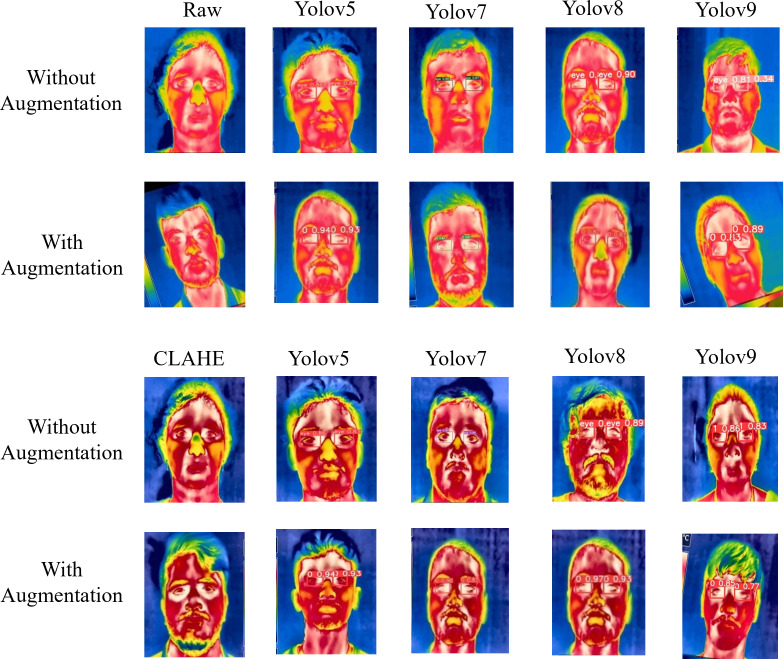
Sample input image and eye-detected image using different Yolo models. A comparative analysis of eye detection on thermal face images using different YOLO models. The first row shows raw images without augmentation, while the second row depicts raw images with augmentation. The third-row displays CLAHE-enhanced images without augmentation, and the fourth row illustrates CLAHE-enhanced images with augmentation. The detection results highlight the impact of augmentation and CLAHE enhancement on improving feature visibility and detection accuracy across different YOLO models.

**Fig 7:**
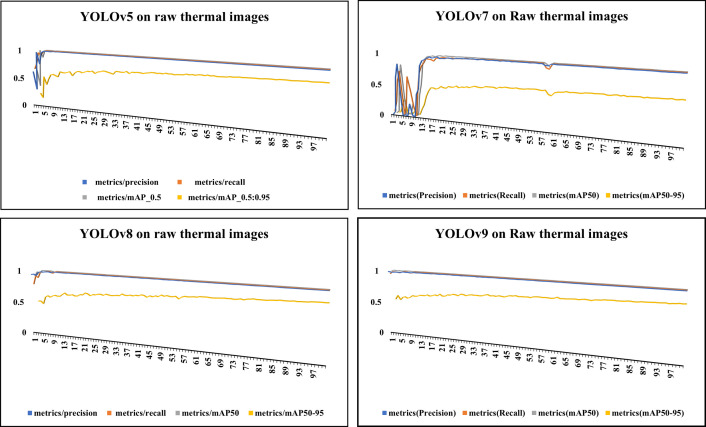
Performance metrics for raw thermal images. This graph shows precision, recall, and mAP variations across different YOLO models trained on raw thermal images.

**Fig 8:**
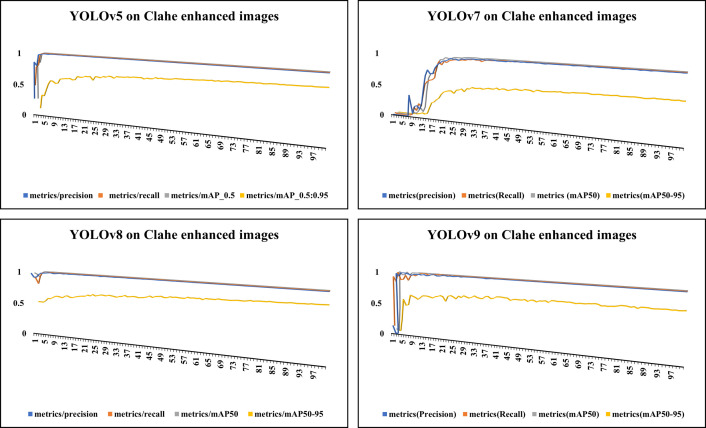
Performance metrics for CLAHE-enhanced thermal images. This graph highlights the improvements in detection accuracy due to contrast enhancement using CLAHE, as measured across YOLO variants.

**Fig 9:**
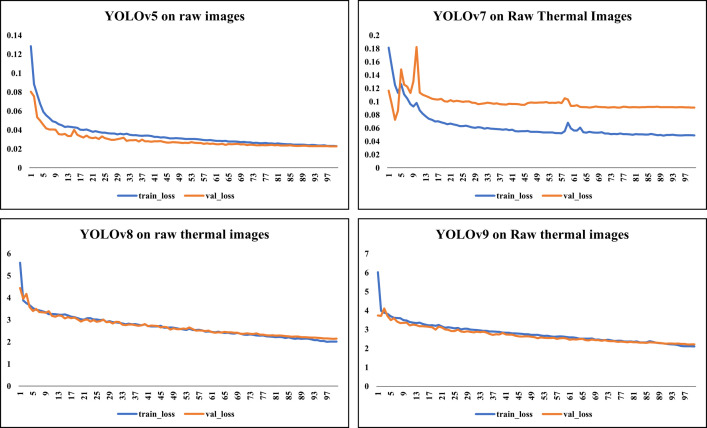
Loss graph for raw thermal images. This figure illustrates training and validation loss convergence across different YOLO models on raw thermal images.

**Fig 10:**
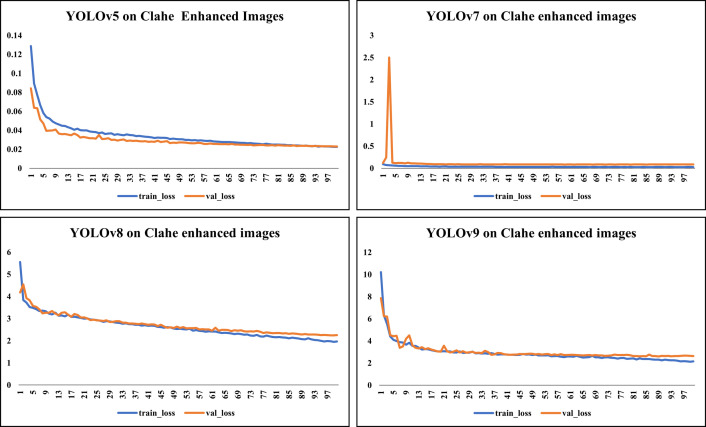
Loss graph for CLAHE-enhanced thermal images. This graph demonstrates the effect of CLAHE preprocessing on model stability and convergence during training.

**Fig 11:**
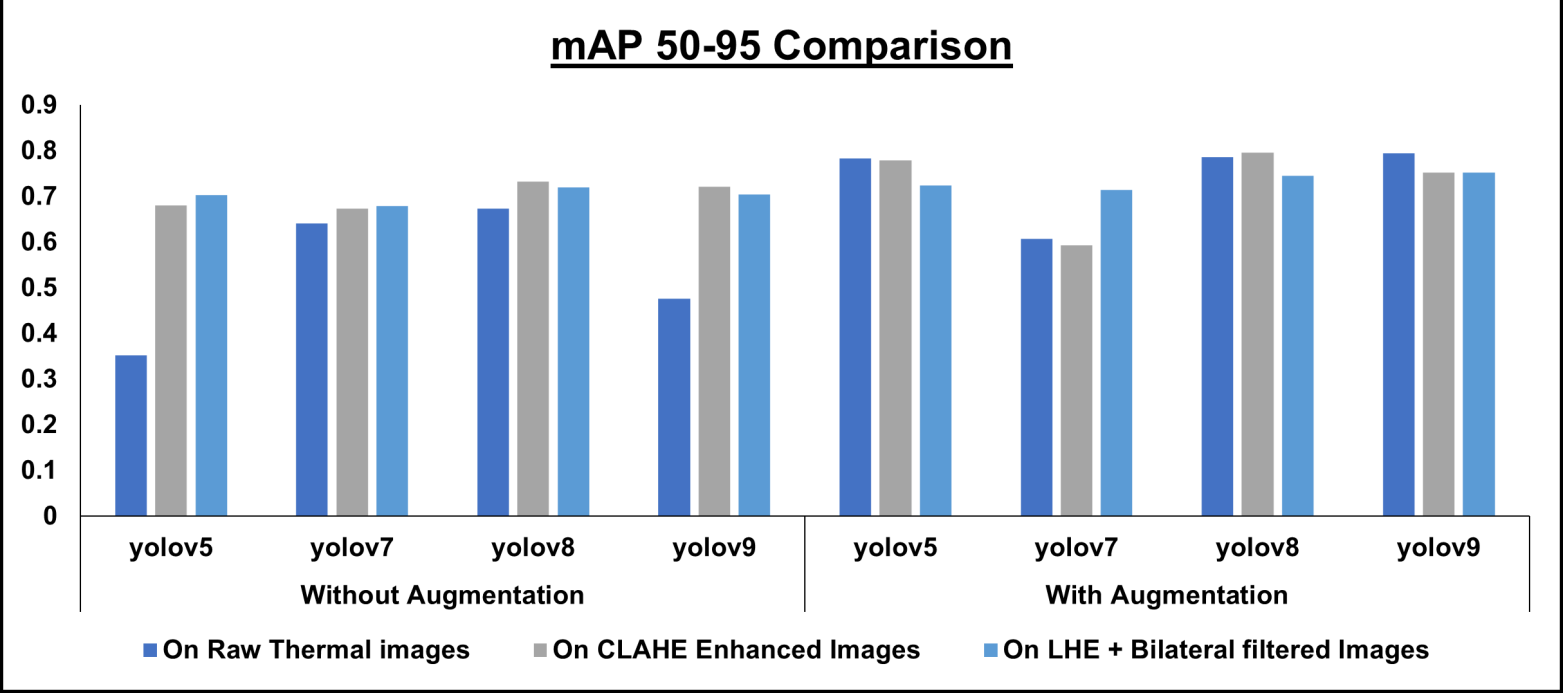
Comparison of mAP50-95 value. Compares mAP50-95 across YOLO variants on raw, CLAHE-enhanced, and LHE + Bilateral filtered thermal images, with and without augmentation. Enhancements and augmentation improve detection accuracy, with YOLOv8 and YOLOv9 achieving the best performance.

Fig 6 showcases sample raw input thermal images and CLAHE-enhanced thermal images and their corresponding eye localization outputs using YOLOv5, YOLOv7, YOLOv8, and YOLOv9. The performance metrics analysed are precision, recall, and mAP metrics and the loss functions obtained by various YOLO variants on augmented dataset are summarized in Figs 7–10 respectively. The loss graph indicates that the YOLOv8 and YOLOv9 models have trained successfully, with both training and validation losses decreasing and stabilizing, which is a sign of good generalization. The bar graph of the comparison between the mAP50-95 metric values produced by several Yolo models using thermal images with and without augmentation is displayed in Fig 11. The graph makes it evident that the YOLOv8 model with augmented images has a better mAP50-95 score.

Different YOLOv8 models, such as YOLOv8n, YOLOv8s, YOLOv8m, YOLOv8l, and YOLOv8x, cater to varying requirements. Selecting the most suitable model depends on the particular requirements. If speed is a critical factor, yolov8n or yolov8s is considered. For scenarios where a good balance between speed and accuracy is needed, yolov8m is a good option. If achieving the highest level of accuracy is the top priority, yolov8l or yolov8x would be the best choices, although it’s important to note that they come with slower inference speeds. Table 5 provides an analysis of different pre-trained YOLOv8 models (yolov8n, yolov8s, yolov8m, yolov8l) on images enhanced with CLAHE both with and without augmentation.

**Table 5:** YOLO8 Performance for different weights.

		Precision	Recall	mAP50	mAP50-95
**Without Augmentation**	Yolov8n.pt	1	1	0.995	0.717
	Yolov8s.pt	0.999	1	0.995	0.72
	Yolov8m.pt	0.991	1	0.995	0.713
	Yolov8l.pt	1	1	0.995	0.721
**With Augmentation**	Yolov8n.pt	1	1	0.995	0.783
	Yolov8s.pt	1	1	0.995	0.796
	Yolov8m.pt	1	1	0.995	0.801
	Yolov8l.pt	1	1	0.995	0.8

*Note:* Compares the performance of YOLOv8 with different pre-trained weights (n, s, m, l) under augmented and non-augmented conditions. All weight variants achieve high precision, recall, and mAP50, while mAP50-95 improves with augmentation, especially for YOLOv8m and YOLOv8l, indicating better feature learning and generalization.

**Fig 12:**
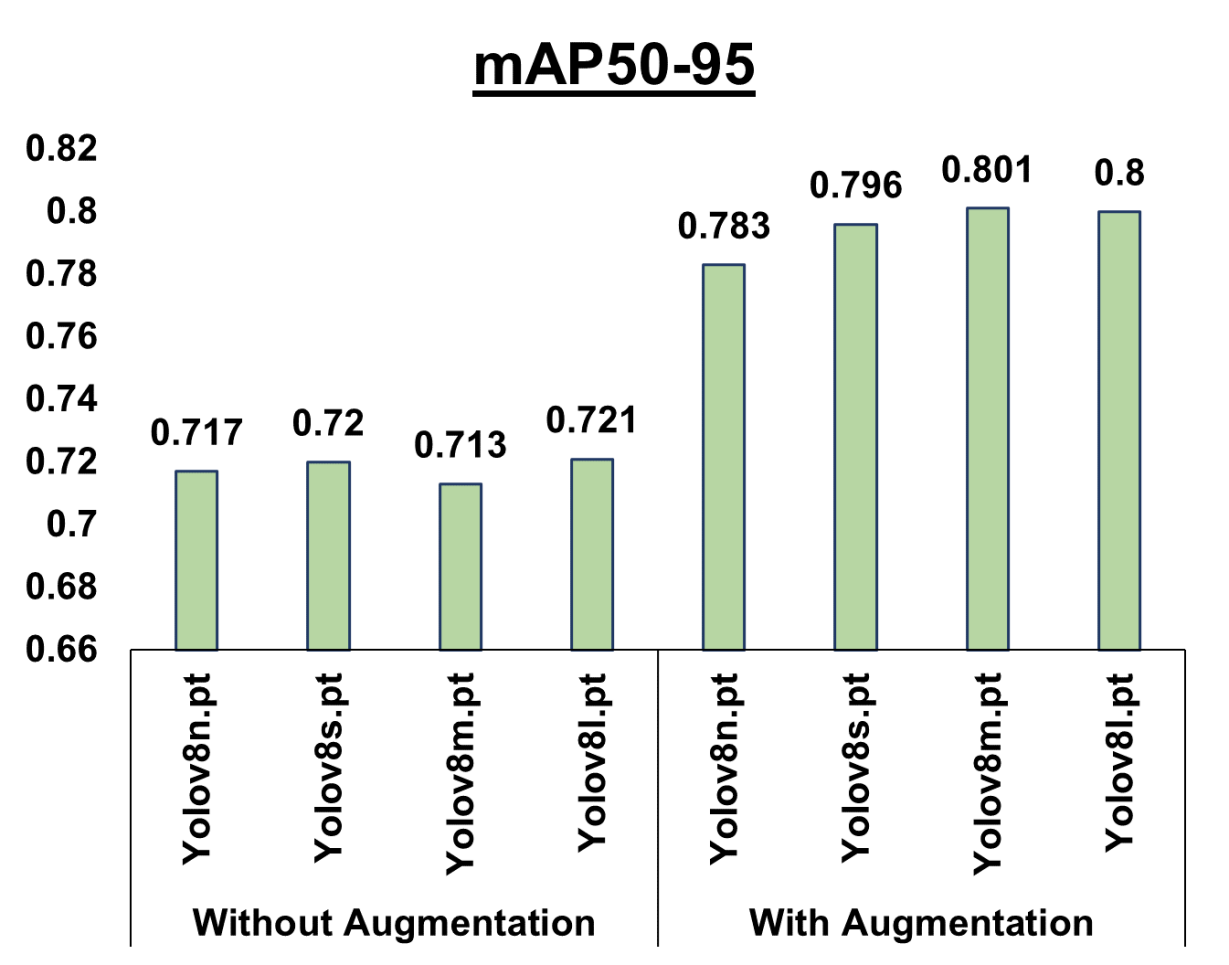
Performance analysis of different weights of YOLOv8 on CLAHE-enhanced images. Compares mAP50-95 for different YOLOv8 weights (n, s, m, l) on CLAHE-enhanced images, with and without augmentation. Augmentation improves accuracy, with YOLOv8m and YOLOv8l performing best.

The metrics such as precision, recall, mAP50, and mAP50-95 indicate that the Yolov8m model performs exceptionally well with augmentation, boasting precision = 1, recall = 1, mAP50 = 0.995, and mAP50-95 = 0.801. Yolov8l closely follows yolov8m, demonstrating comparable performance across all parameters, where yolov8m excels. Other models, such as yolov8n and yolov8s also exhibit good performance, but yolov8m stands out for its superior accuracy. Fig 12 illustrates the comparison of mAP50-95 for CLAHE-enhanced images with and without augmentation. Notably, CLAHE-enhanced images with the yolov8m model achieve the highest accuracy in detecting eyes in thermal images.

Each YOLO model has specific strengths and weaknesses: YOLOv5 is highly precise and performs well with data augmentation, suitable for applications where high precision is critical, but struggles with recall on raw images. YOLOv7 offers balanced performance with high recall, making it reliable for diverse scenarios, though less effective with certain enhancements. YOLOv8 provides strong overall performance and excels with CLAHE-enhanced images, making it versatile but potentially requiring more computational resources. YOLOv9 demonstrates excellent precision and recall with specific enhancements and performs well with augmentation, but its recall on raw images is less robust.

Table 6 provides a clear summary of the descriptive statistics for Precision, Recall, mAP50, and mAP50-95 for various YOLO models, specifically focusing on augmented images. YOLOv8 exhibits the highest Precision and Recall with minimal variability, while YOLOv5 also shows high Precision and Recall but with less consistency compared to YOLOv8. YOLOv7 has the lowest Precision and the highest variability, whereas YOLOv9 displays substantial variability in both Precision and Recall despite having comparable recall values to YOLOv5 and YOLOv8. For mAP50 and mAP50-95, all models perform consistently with identical mean values and very low standard deviations, indicating stable performance across models for these metrics. Hence, our study revealed that YOLOv8 shines in our task requiring both speed and accuracy, making it ideal for real-time applications. This is due to its well-balanced architecture and features like pixel-level segmentation which are particularly useful for detecting small objects like eyes. While YOLOv9 boasts advancements in accuracy through features like PGI and GELAN, YOLOv8’s focus on speed and precision makes it a better choice for our specific task of detecting human eyes in low-resolution thermal images. The precision-recall curve analysis further supports this, which shows YOLOv8 achieving a higher proportion of true positives while minimizing false positives compared to YOLOv9 on our thermal image dataset.

**Table 6:** Descriptive Statistics for the performance of different YOLO models.

	Yolov5		Yolov7		Yolov8		Yolov9	
	Mean	Std_dev	Mean	Std_dev	Mean	Std_dev	Mean	Std_dev
**Precision**	0.998	0.001	0.988	0.014	0.999	0.0009	0.997	1.11
**Recall**	0.997	0.001	0.995	0.003	0.999	0.0004	0.997	1.11
**mAP50**	0.994	0.0009	0.994	0.0009	0.994	0.0009	0.994	0.0009
**mAP50-95**	0.994	0.0009	0.994	0.0009	0.994	0.0009	0.994	0.0009

*Note:* Presents the mean and standard deviation of Precision, Recall, mAP50, and mAP50-95 for YOLOv5, YOLOv7, YOLOv8, and YOLOv9. All models exhibit high consistency and minimal variation, with YOLOv8 achieving the highest precision and recall. The nearly identical mAP50 and mAP50-95 values across models indicate similar detection capabilities with minor performance fluctuations.

The superior performance of the CLAHE and YOLOv8 combination is due to the synergy between enhanced contrast and an advanced detection architecture. CLAHE improves local contrast and enhances edge definition in thermal eye images, which are inherently low in texture and contain subtle intensity variations. This preprocessing step enhances feature visibility. YOLOv8, with its anchor-free detection head, decoupled branches, and CSP-based backbone, effectively detects these refined features, unlike earlier YOLO versions that rely on anchor-based detection, which struggles with low-texture data. In comparison, methods like HE or gamma correction often lose local details. This combination consistently yields higher precision, recall, and mAP than other approaches.

### Comparing super-resolution algorithms through non-reference metrics

**Fig 13:**
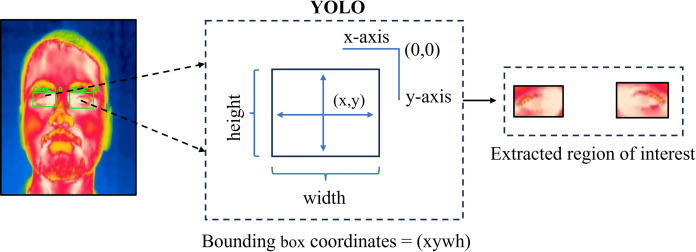
ROI extraction. Illustrates the Region of Interest (ROI) extraction process for thermal eye detection. The left image shows the detected eye region in a thermal face image. The center diagram represents the bounding box expansion around the detected eyes, ensuring optimal coverage for feature extraction. The right image displays the final extracted eye ROIs, which are used for further analysis and processing.

After obtaining the eye-detected output from the trained and validated YOLO models, the detected bounding box coordinates are used to crop the detected eye regions for further analysis. The ROI extraction is shown in Fig 13. However, the extracted eye regions often suffer from low resolution. Implementing super-resolution on the whole thermal image escalates its complexity. So, we only apply super-resolution to the cropped area around the eyes (ROI) which significantly reduces complexity while improving the quality of the specific area we care about. Hence to address this issue and improve the resolution, various super-resolution algorithms are applied, including bicubic interpolation, BSRGAN, ESRGAN, real-ESRGAN, swinIR, swinIR-Large, and ResShift. In our study, we employed various super-resolution techniques to enhance the resolution of the cropped eye region from thermal face images by a factor of x4. All methods utilized the authors’ pre-trained models without any modification to hyperparameters, which is shown in Table 7. Each technique adhered to default settings and configurations, ensuring a consistent evaluation framework across all methods. This approach allowed us to leverage established model architectures and training regimes for a robust comparison of super-resolution performance. Table 8 presents the performance analysis of these different super-resolution algorithms, evaluated using non-reference metrics such as NIQE, BRISQUE, and PIQE.

**Table 7:** Hyperparameters Used by Different Super-Resolution Models.

Algorithm	Learning Rate	Batch Size	Optimizer	Loss Function
Bicubic	NA	NA	NA	NA
BSRGAN	1e-5	16	Adam	L1 loss, GAN loss
ESRGAN	1e-4	16	Adam	Perceptual loss, GAN loss
Real-ESRGAN	1e-4	16	Adam	Perceptual loss, GAN loss
SwinIR	2e-4	32	Adam	L1 loss
SwinIR-Large	2e-4	32	Adam	L1 loss
ResShift	1e-3	16	Adam	L2 loss

*Note:* Summarizes the learning rate, batch size, optimizer, and loss function used by various super-resolution models. Traditional Bicubic interpolation does not require these parameters, while deep learning-based models (BSRGAN, ESRGAN, Real-ESRGAN, SwinIR, SwinIR-Large, and ResShift) employ Adam optimization with different loss functions such as L1, L2, perceptual loss, and GAN loss. SwinIR models use larger batch sizes and higher learning rates, while ResShift adopts an L2 loss function with the highest learning rate among the models.

**Table 8:** Performance Analysis of Super-resolution Algorithms.

On Raw Images															
	Image 1			Image 2			Image 3			Image 4			Image 5		
Techniques	NIQE	BRISQUE	PIQE	NIQE	BRISQUE	PIQE	NIQE	BRISQUE	PIQE	NIQE	BRISQUE	PIQE	NIQE	BRISQUE	PIQE
Bicubic interpolation	6.13	62.51	100	6.17	61.29	100	6.48	64.85	100	6.05	63.26	100	6.72	61.87	100
BSRGAN	5.44	37.35	38.96	5.13	40.68	37.18	5.47	39.43	48.27	5.36	39.84	37.28	5.91	45.18	36.93
ESRGAN	5.33	22.66	8.62	4.69	27.25	14.61	4.40	34.49	40.47	4.17	28.18	25.54	4.29	34.13	19.57
Real-ESRGAN	4.46	49.84	30.32	3.53	50.57	34.46	4.98	50.30	39.58	5.06	50.97	57.55	5.27	58.27	58.76
SwinIR	5.66	43.21	42.00	5.03	43.66	49.85	5.34	45.57	53.02	4.78	42.40	45.73	4.44	46.83	32.63
SwinIR-Large	5.50	41.45	52.89	4.45	48.47	65.49	4.48	42.09	53.67	6.01	40.04	41.38	6.79	54.33	73.59
ResShift	5.30	32.78	24.86	6.13	46.64	36.76	5.49	46.57	28.84	4.79	27.09	21.60	4.53	27.79	17.03
**On CLAHE images**															
	**Image 1**			**Image 2**			**Image 3**			**Image 4**			**Image 5**		
**Techniques**	**NIQE**	**BRISQUE**	**PIQE**	**NIQE**	**BRISQUE**	**PIQE**	**NIQE**	**BRISQUE**	**PIQE**	**NIQE**	**BRISQUE**	**PIQE**	**NIQE**	**BRISQUE**	**PIQE**
Bicubic interpolation	5.74	57.81	100	6.46	63.27	100	5.74	58.50	100	5.92	61.06	100	6.75	55.30	100
BSRGAN	5.12	42.88	52.73	5.03	37.86	35.87	6.14	37.63	37.28	4.46	38.98	34.14	5.82	47.84	45.01
ESRGAN	5.20	23.78	19.61	5.46	30.35	18.49	4.17	10.44	17.80	7.67	30.02	18.29	7.72	27.35	18.05
Real-ESRGAN	4.56	55.39	69.58	5.35	51.34	34.25	6.46	53.81	68.99	3.65	48.37	43.01	5.47	56.23	65.57
SwinIR	5.41	44.03	63.12	4.40	40.89	30.48	5.87	46.06	39.44	4.46	49.13	43.35	4.97	49.24	57.35
SwinIR-Large	5.77	50.16	90.96	5.53	50.15	57.16	5.65	50.81	65.70	4.90	50.47	51.05	4.58	48.45	55.39
ResShift	8.70	53.63	47.92	4.89	49.92	45.09	6.46	47.22	58.12	5.92	39.32	13.50	5.64	50.14	62.08

*Note:* Performance comparison of various super-resolution algorithms applied to raw and CLAHE-enhanced images, evaluated using NIQE, BRISQUE, and PIQE metrics across sample images. Lower values in these metrics indicate better image quality. Bicubic interpolation consistently yields the highest PIQE scores, signifying poor perceptual quality. ESRGAN and BSRGAN demonstrate superior performance, achieving lower BRISQUE and PIQE values, indicating enhanced image sharpness and perceptual quality. CLAHE processing improves NIQE scores in some cases. Real-ESRGAN and ResShift offer a balanced approach, optimizing perceptual and objective quality metrics. These results highlight the varying effectiveness of super-resolution techniques in enhancing thermal images.

**Fig 14:**
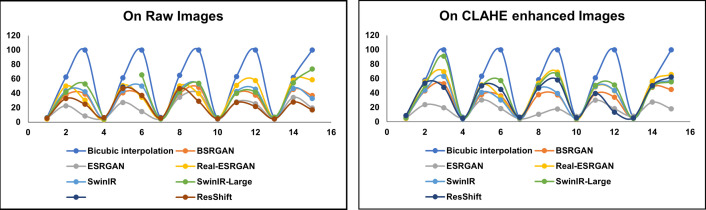
Scatter Plot of non-reference metrics for a few sample images. Compares the non-reference image quality metrics (NIQE, BRISQUE, and PIQE) for different super-resolution techniques on raw and CLAHE-enhanced images. The left plot represents raw images, while the right plot shows CLAHE-enhanced images. The results highlight the impact of CLAHE processing on improving image quality and the effectiveness of deep learning-based super-resolution methods over traditional interpolation techniques.

**Fig 15:**
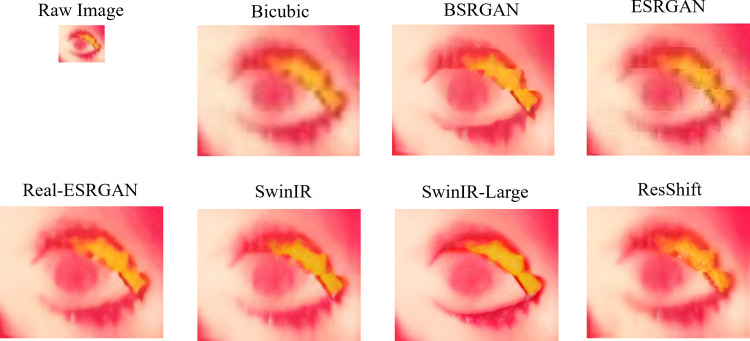
Sample raw image vs. Super-Resolution Results. Compares a raw thermal eye image with outputs from different super-resolution techniques, highlighting improvements in clarity, edge sharpness, and thermal feature preservation for better analysis.

**Fig 16:**
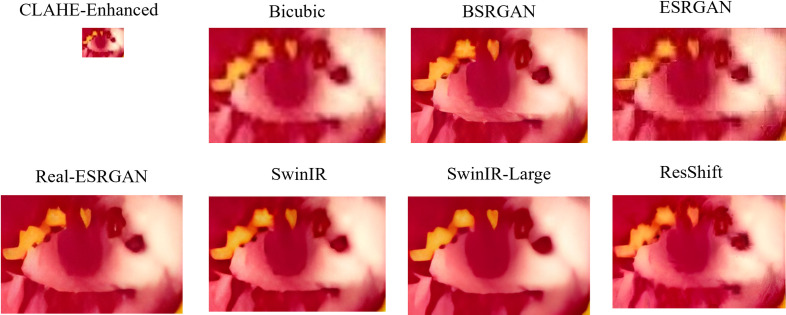
Sample CLAHE enhanced image vs. Super-Resolution Results. Presents a CLAHE-enhanced thermal eye image (top-left) alongside outputs from various super-resolution techniques. The results highlight the combined effect of contrast enhancement and high-resolution reconstruction, aiding in more precise thermal image analysis.

The performance of each image resolution enhancement technique is assessed by comparing the values across NIQE, BRISQUE, and PIQE metrics, with lower values generally indicating better image quality. Fig 14 illustrates the scatter plot of the non-reference metrics for a few samples of raw images and the enhanced images. It clearly illustrates bicubic interpolation has elevated scores suggesting limitations in achieving substantial improvements. BSRGAN demonstrates competitive performance across various image types, exhibiting minor variations in scores. In assessing image enhancement techniques based on naturalness and spatial quality metrics such as NIQE and BRISQUE, ESRGAN consistently demonstrates strong performance with consistently low scores. This suggests that ESRGAN excels in maintaining the natural appearance of images and preserving spatial details effectively. On the other hand, when prioritizing perceptual quality, as measured by PIQE, ESRGAN again excels and the Real-ESRGAN, SwinIR, SwinIR-Large, and ResShift emerge as competitive options. Figs 15 and 16 visualize the output of different super-resolution algorithms.

### Subjective assessment of images enhanced through super-resolution

Non-reference metrics like PIQE, BRISQUE, and NIQE prioritize statistical features over human visual preferences and context-specific factors. Consequently, these metrics do not fully capture the intricacies of human visual perception and preferences, leading to potential discrepancies between the metric scores and human perceptual judgments [44]. Objective and subjective quality assessment methods don’t always exhibit a strong correlation with each other [45]. Therefore, subjective assessment of images enhanced through super-resolution is essential. It helps evaluate perceived improvements in quality and provides insights into the effectiveness of different algorithms and their impact on visual perception. This aspect cannot be fully captured by objective metrics. In our study, subjective image assessment is conducted using the single stimulus method, wherein images are presented individually to participants who rated their quality before moving on to the next image [46]. This method, employs the Absolute Category Rating scale in which the subject is bound to grade the image quality on a scale of five points, which are: bad, poor, fair, good, and excellent [47]. Table 9 shows the scale of image quality grade used and the score assigned to each. It simplifies the assessment process but may prolong testing time with a large image set. Factors such as image content influence participants’ opinions, and mean opinion scores (MOS) are calculated based on their feedback [48]. The formula for calculating MOS is expressed in Equation 17.

**Table 9:** Five Grade Scale.

Grade	Score
Bad	1
Poor	2
Fair	3
Good	4
excellent	5

*Note:* The table defines a five-point grading scale used for evaluation. Scores range from 1 (Bad) to 5 (Excellent), providing a standardized assessment framework for qualitative analysis.


$$MOS=\;\frac{1}{N}\sum_{i}^{N}x_{i}$$ (17)

Where $\sum_{i}^{N}x_{i}\;$ represents the sum of all individual scores provided by subjects. $\frac{1}{N}$ calculates the average by dividing the sum by the total number of subjects.

**Fig 17:**
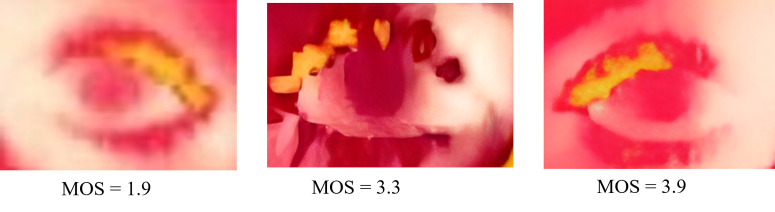
Sample Images with MOS value. Sample images with their assigned MOS based on a five-point scale, assessing quality in terms of sharpness, clarity, and feature preservation. The ratings offer an objective comparison of super-resolution performance across different methods.

**Fig 18:**
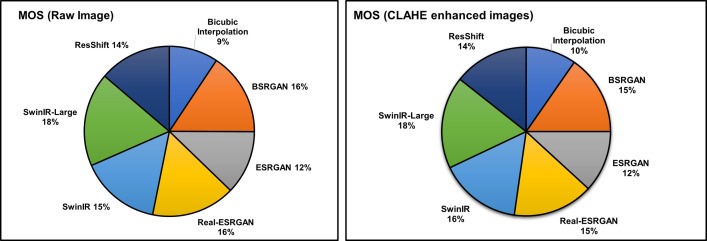
Pie chart illustrating the comparison of Mean Opinion Scores. Compares MOS for super-resolution techniques on raw and CLAHE-enhanced images. SwinIR-Large achieves the highest MOS (18%), indicating superior perceptual quality, while Bicubic interpolation scores the lowest. CLAHE enhancement improves MOS for most methods, with BSRGAN, Real-ESRGAN, and SwinIR showing notable gains.

In compliance with ITU-R BT.500−12 recommendations [49], we conduct tests in a controlled environment using a 4K resolution monitor. Our participant pool consists mostly of research students who are familiar with multimedia applications and image quality, totaling 20 individuals. They received a briefing on the test procedure before evaluating a total of 15 images, with 5 from each category: raw thermal extracted eye images, CLAHE enhanced eye images, and LHE with bilateral filtering eye images. Each participant repeated the scoring five times, and the MOS was calculated based on these repeated assessments. In light of the display size, participants are urged to perform the exam in a leisurely manner without regard to time limitations and are permitted to sit at their desired comfortable viewing distance. We record participants’ scores for different super-resolution algorithms applied to the test images, which are used to calculate the MOS for each technique. The consistent trends observed across repetitions ensured the reliability of the subjective evaluation. Fig 17 depicts sample images along with their corresponding calculated MOS values. Our findings indicate comparable performance among BSRGAN, Real-ESRGAN, SwinIR, SwinIR-Large, and ResShift, surpassing that of ESRGAN. The summary of MOS calculations is presented in Table 10, with a pie chart in Fig 18 illustrating the mean MOS values. SwinIR-Large demonstrates strong performance, while other techniques such as BSRGAN, Real-ESRGAN, SwinIR, and ResShift also perform well with slight variations.

**Table 10:** Summary MOS Calculated.

Techniques	MOS (Raw Image)				
**Image_1**	**Image_2**	**Image_3**	**Image_4**	**Image_5**
Bicubic Interpolation	1.8	1.9	1.9	2.2	2.1
BSRGAN	3.0	3.5	3.3	3.4	3.5
ESRGAN	2.2	2.6	3.3	2.5	2.3
Real-ESRGAN	2.9	3.6	3.4	3.4	3.6
SwinIR	2.8	3.5	3.1	3.3	3.5
SwinIR-Large	3.4	4.4	3.8	3.9	3.4
ResShift	2.7	3.0	3.0	3.1	2.8
**Techniques**	**MOS (CLAHE enhanced images)**				
	**Image_1**	**Image_2**	**Image_3**	**Image_4**	**Image_5**
Bicubic Interpolation	2.2	2.0	2.3	2.1	2.1
BSRGAN	3.1	3.3	3.6	3.6	3.4
ESRGAN	2.7	2.6	2.5	2.7	2.6
Real-ESRGAN	3.5	3.4	3.4	3.5	3.3
SwinIR	3.7	3.4	3.4	3.4	3.5
SwinIR-Large	3.8	4.1	4.0	3.9	3.8
ResShift	3.2	3.2	3.2	3.1	3.2

*Note:* Summarizes the Mean Opinion Score (MOS) for various super-resolution methods on raw and CLAHE-enhanced images. MOS is rated on a 1–5 scale, with higher scores indicating better quality.

**Fig 19:**
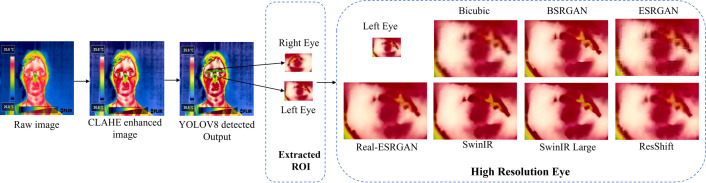
Complete Flow of Results of Thermal Image Processing for Human Eye Detection and Enhancement. Illustrates the workflow of thermal eye detection and super-resolution enhancement. The left section shows input thermal images and ROI extraction for the left and right eyes, while the right section compares super-resolution results using various techniques. Deep learning-based methods significantly improve clarity and feature preservation over traditional interpolation.

Fig 19 illustrates the complete workflow from the initial raw thermal image through the stages of contrast enhancement, eye region detection, extraction, and super-resolution processing. The choice of a super-resolution algorithm involves balancing image quality against computational complexity. Traditional methods like Bicubic Interpolation offer quick and resource-efficient solutions at the expense of finer details, while advanced techniques like ESRGAN, Real-ESRGAN, and SwinIR provide superior image quality with higher computational costs. While ResShift is less demanding than GAN-based methods, it still requires considerable computational resources due to its deep network architecture. Understanding these trade-offs is crucial for selecting the appropriate method based on specific application requirements and available computational resources.

## Practical Implication and Potential Application

The proposed technology for accurate eye detection in thermal images has significant potential in various real-world applications. In biometrics, it enhances facial and iris recognition systems, improving security in low-light conditions. In healthcare, it facilitates patient monitoring, ophthalmology, and telemedicine by accurately tracking vital signs, ocular temperature, and stress levels through thermal imaging. For driver monitoring, it enables the detection of drowsiness and distraction, thereby enhancing road safety. In human-computer interaction, precise eye detection can be used for gaze tracking and emotion recognition, improving user experience and accessibility. This technology offers robust solutions across multiple fields where traditional imaging methods fall short due to poor lighting or visibility conditions. Implementing the proposed eye detection and enhancement pipeline in real-time systems like driver monitoring or biometric authentication is feasible but challenging. It requires high-performance hardware (e.g., GPUs, AI accelerators) and optimization techniques (e.g., model pruning, quantization, parallel processing) to handle the computational load and reduce latency. Additionally, integrating quality thermal cameras and ensuring robust performance under varying conditions is crucial. Reliable and efficient deployment is achievable with appropriate hardware, optimization, and extensive real-world testing.

While these technological advancements are significant, it is crucial to address the ethical considerations associated with the use of eye detection technology in sensitive thermal applications. To protect privacy, informed consent, anonymization, and data minimization are essential. Transparent communication and voluntary participation ensure proper consent. Data security should be maintained and to avoid bias, diverse datasets are necessary. The potential misuse of this technology for increased surveillance or discriminatory practices must be mitigated through ethical guidelines. Addressing these ethical considerations is essential for the responsible and ethical use of eye detection technology in thermal applications.

## Limitations and Future Improvement

Super-resolution techniques are effective but can introduce artifacts. Evaluation using non-reference metrics and subjective mean opinion scores may lack objectivity, and pre-trained models may carry inherent biases. While PIQE, BRISQUE, and NIQE provide insights into thermal image quality, they were designed for natural images. Future work can develop task-specific non-reference image quality assessment metrics methods for more accurate super-resolution assessment in thermal imaging. Deep learning-based methods are computationally demanding, limiting their suitability for real-time applications. Improvements could include adaptive pre-processing to enhance thermal image quality, fine-tuning detection models with a more diverse range of datasets, developing hybrid super-resolution methods that optimize both accuracy and computational efficiency, and conducting extensive real-world testing. The thermal images collected provide temperature data for the detected eye region, but a more diverse dataset—capturing variations in eye conditions, geographic demographics, race, and age groups—is needed. This would support applications in healthcare and ophthalmology. Such advancements can improve the robustness, efficiency, and fairness of the approach, thereby expanding its potential uses.

## Conclusion

This study presents an innovative approach that revolutionizes eye detection and resolution enhancement in thermal facial images by integrating deep learning (particularly YOLOv8 and YOLOv9), image enhancement (CLAHE), and super-resolution techniques. This unique blend achieves unmatched accuracy (precision and recall of 1, mAP50 of 0.995) even in challenging low-resolution and low-contrast thermal data. The key contributions include the development of a pioneering pipeline seamlessly combining CLAHE pre-processing, YOLO-based eye detection, and super-resolution techniques. Also, a unique dataset of thermal facial images with meticulously labeled eye locations is introduced. While YOLOv8 currently excels, further exploration of YOLOv9’s potential through ablation studies is warranted. Future research directions include exploring multimodal biometric systems, advanced deep learning architectures, continuous learning strategies, and robust super-resolution algorithms tailored for thermal images, aiming to further enhance eye detection capabilities and unlock its full potential in various domains.

## Supporting information

S1 File This file contains Supplementary Figures S1-S25. (ZIP)
