# Supplementary figures and images for: Synergistic fusion: An integrated pipeline of CLAHE, YOLO models, and advanced super-resolution for enhanced thermal eye detection
Source: PLoS One. 2025 Jul 18;20(7):e0328227. doi: 10.1371/journal.pone.0328227 (PMC12273955; doi:10.1371/journal.pone.0328227)

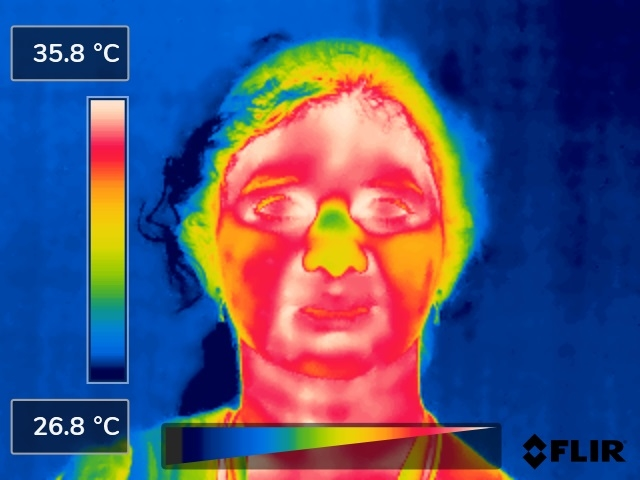

Supplement: S1 File — (ZIP) [file pone.0328227.s001.zip › PLOS_SI/S1.tif]

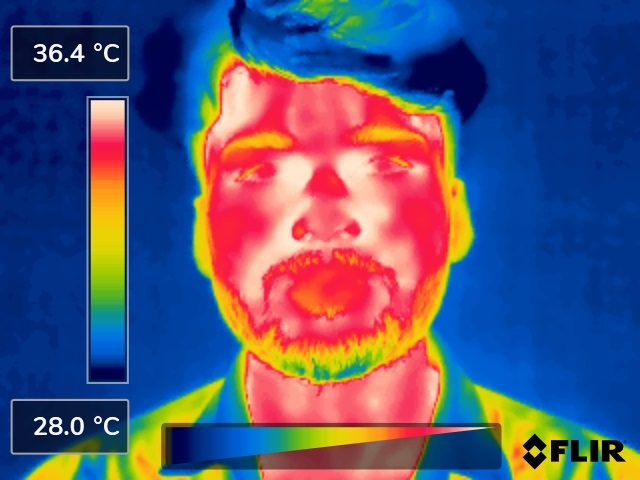

Supplement: S1 File — (ZIP) [file pone.0328227.s001.zip › PLOS_SI/S10.tif]

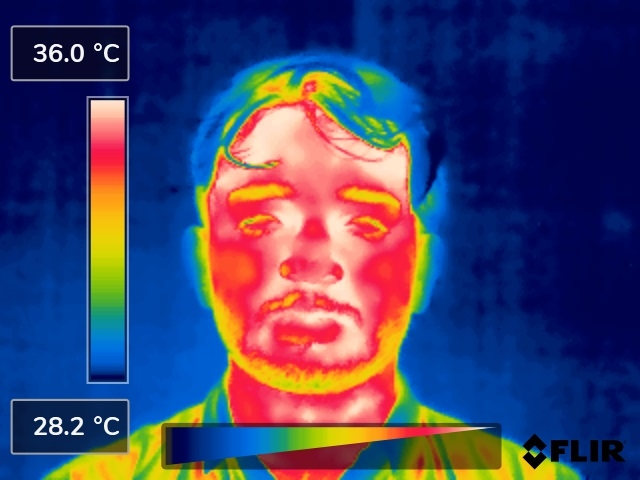

Supplement: S1 File — (ZIP) [file pone.0328227.s001.zip › PLOS_SI/S11.tif]

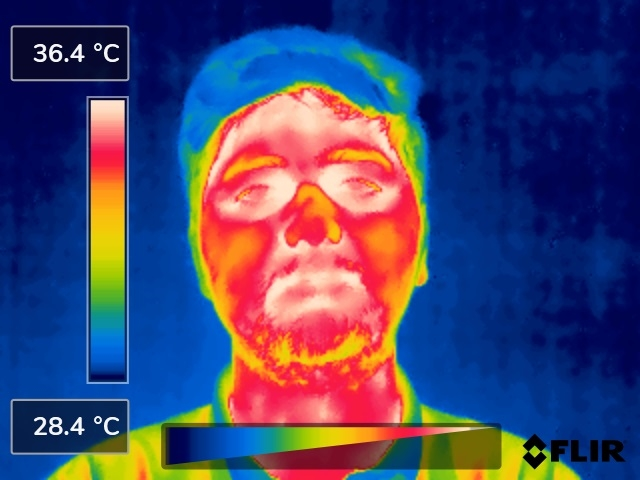

Supplement: S1 File — (ZIP) [file pone.0328227.s001.zip › PLOS_SI/S12.tif]

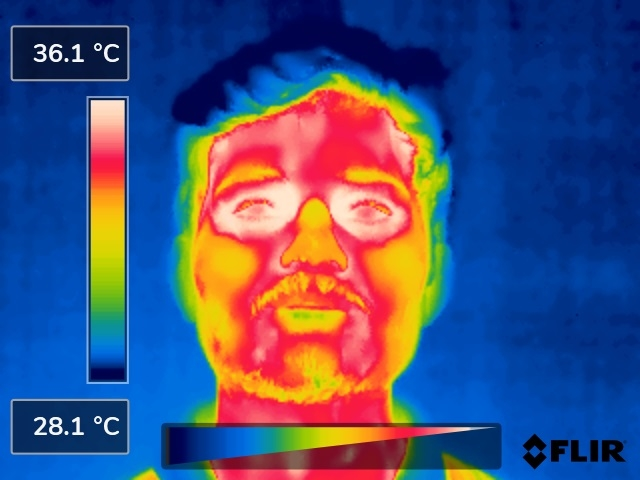

Supplement: S1 File — (ZIP) [file pone.0328227.s001.zip › PLOS_SI/S13.tif]

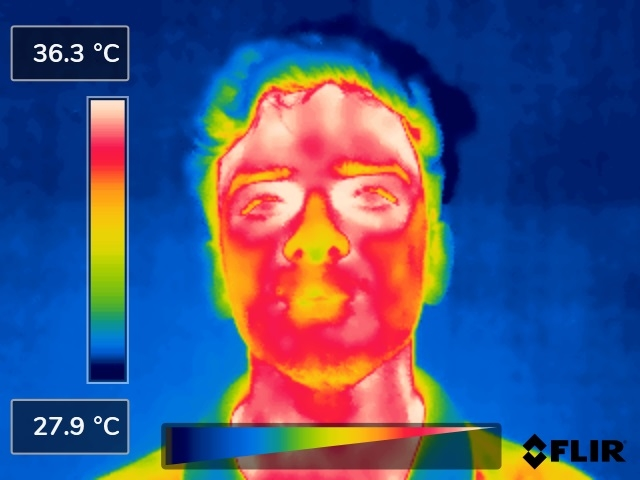

Supplement: S1 File — (ZIP) [file pone.0328227.s001.zip › PLOS_SI/S14.tif]

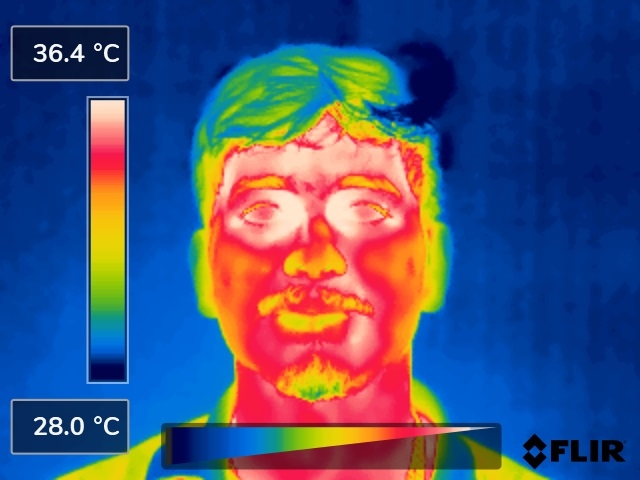

Supplement: S1 File — (ZIP) [file pone.0328227.s001.zip › PLOS_SI/S15.tif]

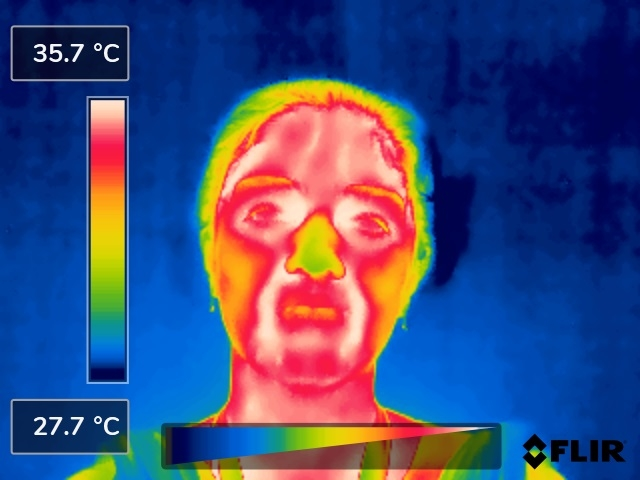

Supplement: S1 File — (ZIP) [file pone.0328227.s001.zip › PLOS_SI/S16.tif]

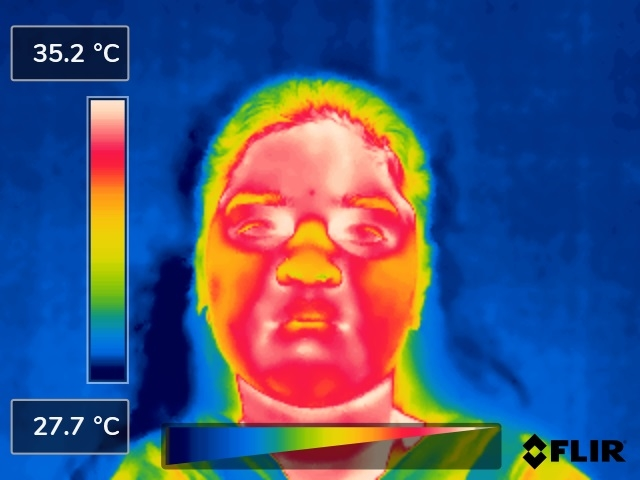

Supplement: S1 File — (ZIP) [file pone.0328227.s001.zip › PLOS_SI/S17.tif]

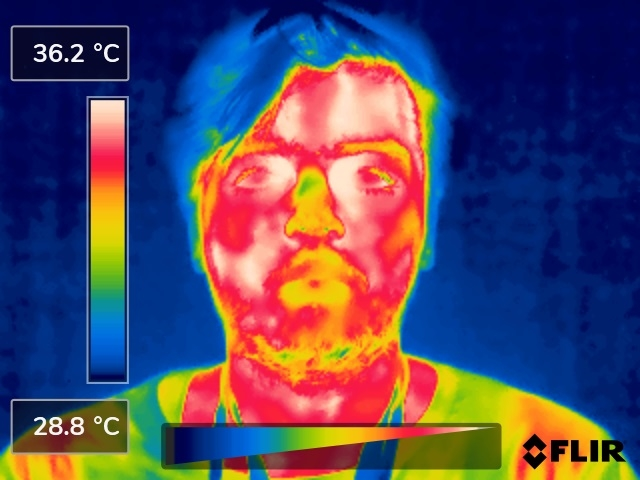

Supplement: S1 File — (ZIP) [file pone.0328227.s001.zip › PLOS_SI/S18.tif]

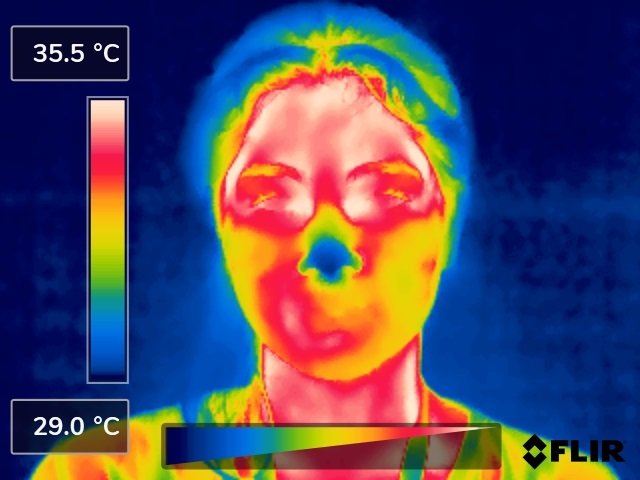

Supplement: S1 File — (ZIP) [file pone.0328227.s001.zip › PLOS_SI/S19.tif]

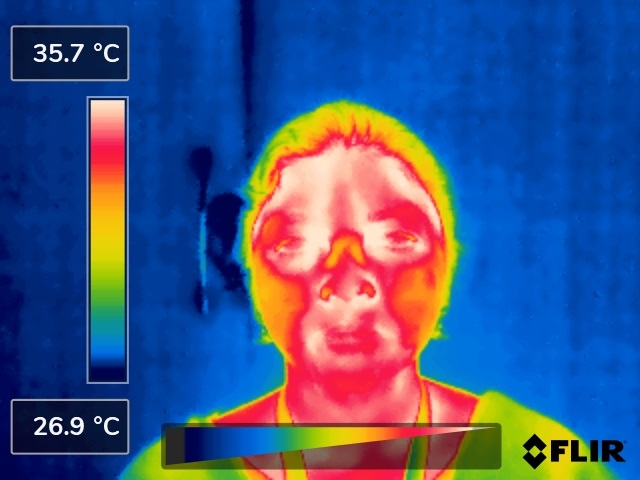

Supplement: S1 File — (ZIP) [file pone.0328227.s001.zip › PLOS_SI/S2.tif]

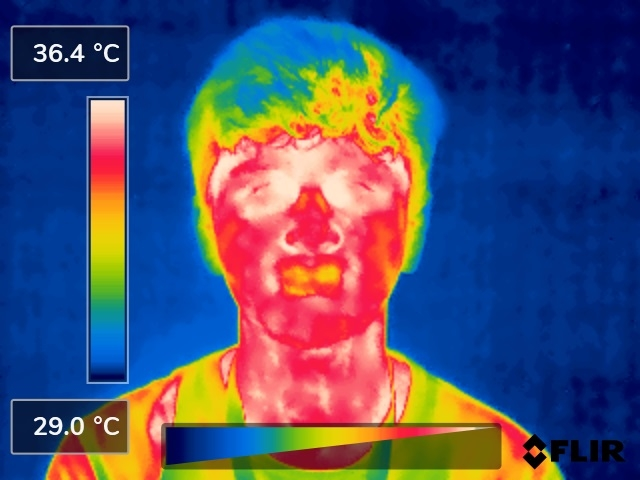

Supplement: S1 File — (ZIP) [file pone.0328227.s001.zip › PLOS_SI/S20.tif]

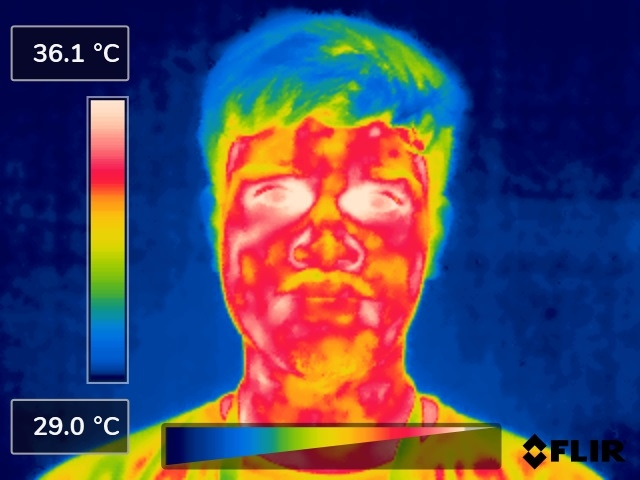

Supplement: S1 File — (ZIP) [file pone.0328227.s001.zip › PLOS_SI/S21.tif]

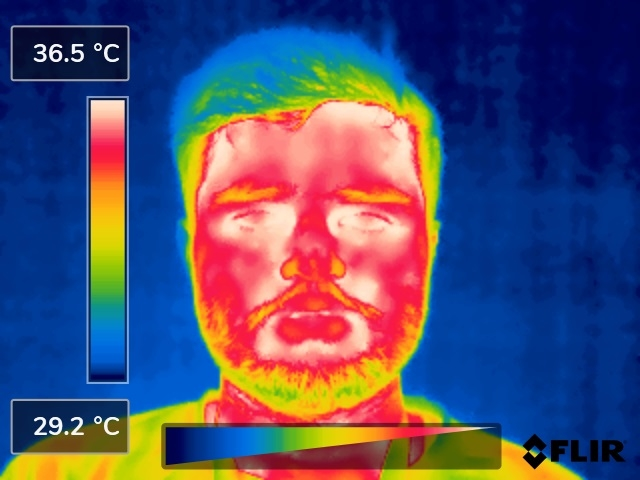

Supplement: S1 File — (ZIP) [file pone.0328227.s001.zip › PLOS_SI/S22.tif]

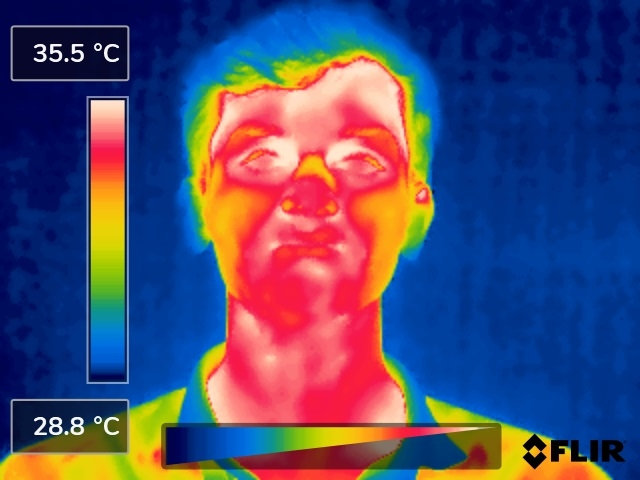

Supplement: S1 File — (ZIP) [file pone.0328227.s001.zip › PLOS_SI/S23.tif]

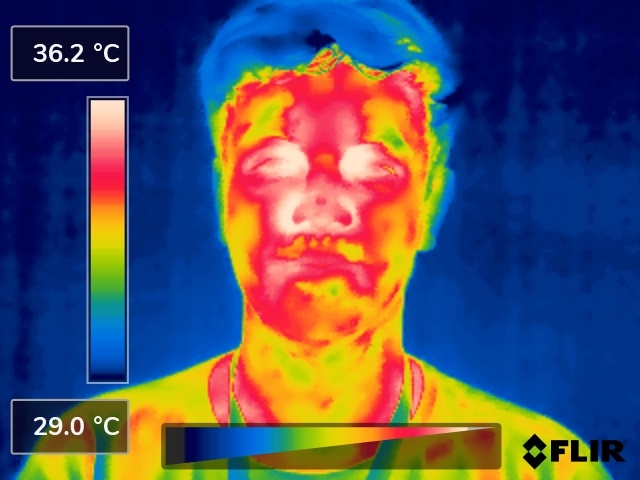

Supplement: S1 File — (ZIP) [file pone.0328227.s001.zip › PLOS_SI/S24.tif]

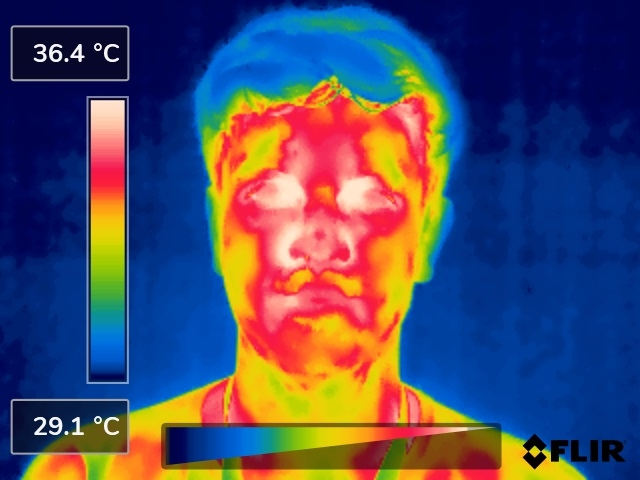

Supplement: S1 File — (ZIP) [file pone.0328227.s001.zip › PLOS_SI/S25.tif]

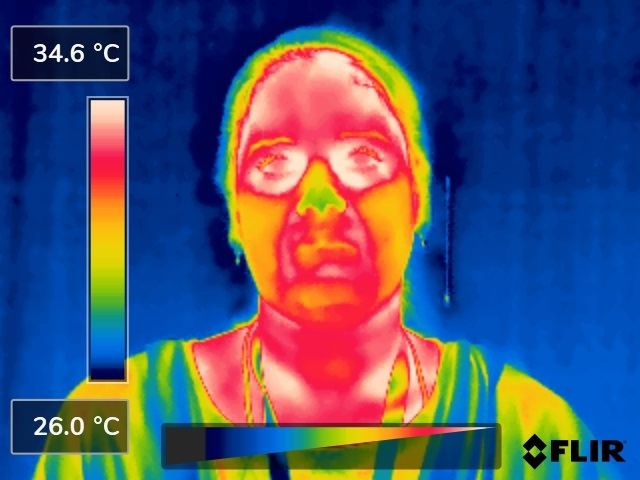

Supplement: S1 File — (ZIP) [file pone.0328227.s001.zip › PLOS_SI/S3.tif]

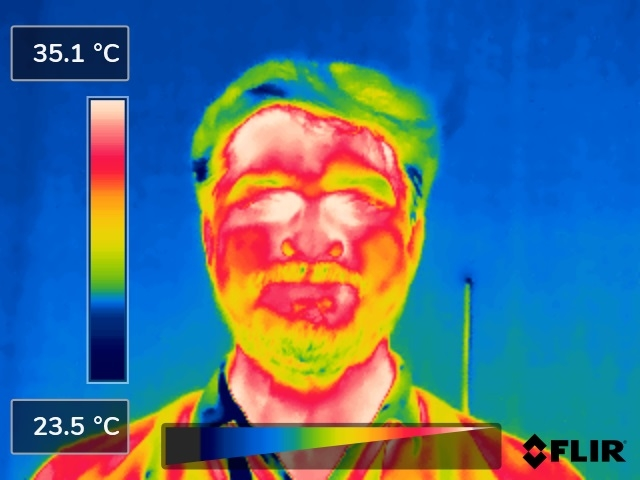

Supplement: S1 File — (ZIP) [file pone.0328227.s001.zip › PLOS_SI/S4.tif]

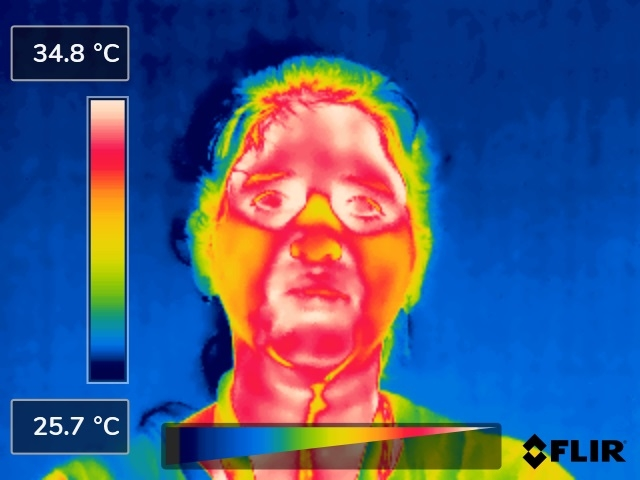

Supplement: S1 File — (ZIP) [file pone.0328227.s001.zip › PLOS_SI/S5.tif]

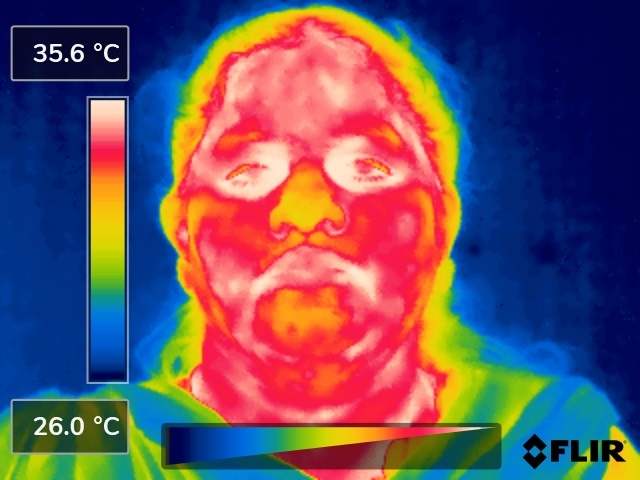

Supplement: S1 File — (ZIP) [file pone.0328227.s001.zip › PLOS_SI/S6.tif]

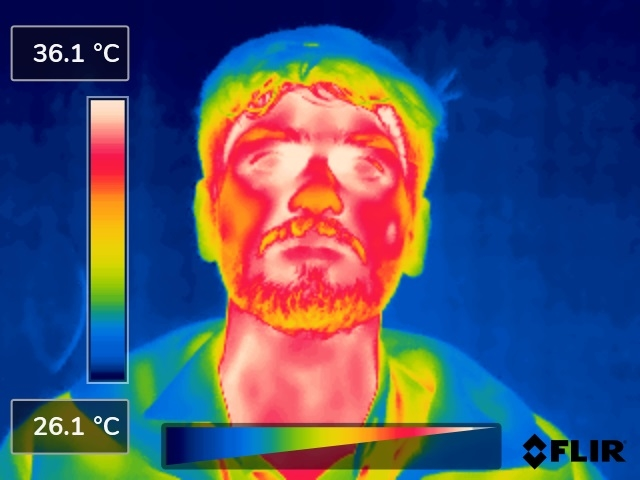

Supplement: S1 File — (ZIP) [file pone.0328227.s001.zip › PLOS_SI/S7.tif]

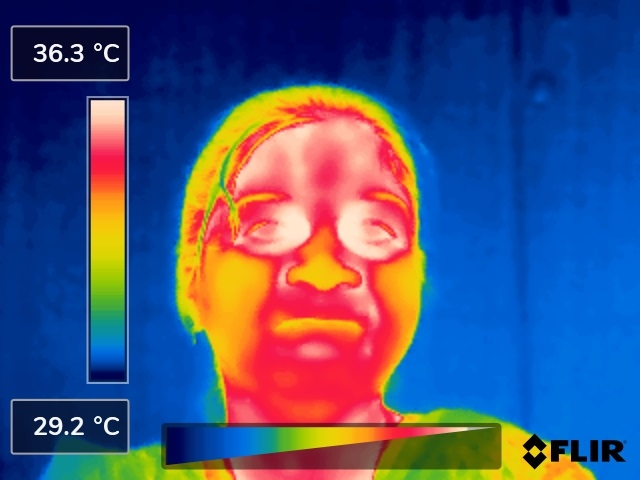

Supplement: S1 File — (ZIP) [file pone.0328227.s001.zip › PLOS_SI/S8.tif]

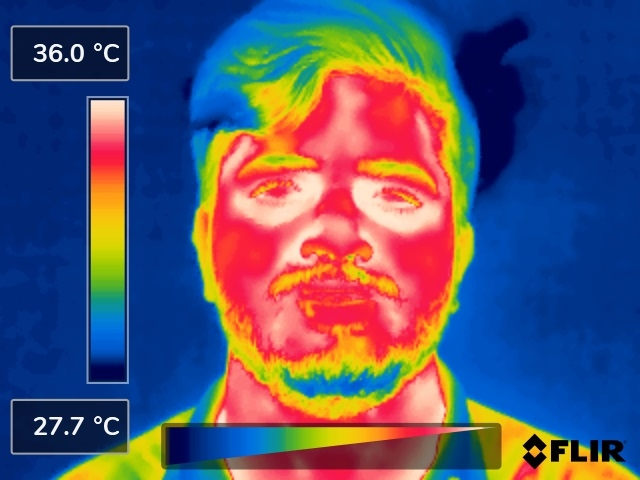

Supplement: S1 File — (ZIP) [file pone.0328227.s001.zip › PLOS_SI/S9.tif]
